# Enhanced CTLA‐4 blockade anti‐tumor immunity with APG‐157 combination in a murine head and neck cancer

**DOI:** 10.1002/cam4.7212

**Published:** 2024-04-30

**Authors:** Daniel Sanghoon Shin, Saroj Basak, Mysore S. Veena, Begoña Comin‐Anduix, Arjun Bhattacharya, Tien S. Dong, Albert Ko, Philip Han, Jonathan Jacobs, Neda A. Moatamed, Luis Avila, Matteo Pellegrini, Marilene Wang, Eri S. Srivatsan

**Affiliations:** ^1^ Department of Medicine, Division of Hematology‐Oncology VAGLAHS/David Geffen School of Medicine at University of California, Los Angeles (UCLA) Los Angeles California USA; ^2^ Department of Surgery David Geffen School of Medicine at UCLA Los Angeles California USA; ^3^ Parker Institute for Cancer Immunotherapy Los Angeles California USA; ^4^ Ahmanson Translational Theranostics Division, Department of Molecular and Medical Pharmacology UCLA Los Angeles, California USA; ^5^ Department of Chemistry and Biochemistry and the Institute for Quantitative and Computational Biology UCLA Los Angeles California USA; ^6^ Department of Louise M. Darling Biomedical Library and The Institute for Quantitative and Computational Biology UCLA Los Angeles California USA; ^7^ Division of Digestive Diseases, Department of Medicine David Geffen School of Medicine at UCLA Los Angeles California USA; ^8^ Department of Pathology and Laboratory Medicine David Geffen School of Medicine at UCLA Los Angeles California USA; ^9^ Department of Molecular Cellular and Developmental Biology, UCLA Los Angeles California USA; ^10^ Department of Surgery, VAGLAHS and Department of Head and Neck Surgery David Geffen School of Medicine at UCLA Los Angeles California USA; ^11^ Department of Surgery VAGLAHS/David Geffen School of Medicine at UCLA Los Angeles California USA; ^12^ Present address: Department of Epidemiology The University of Texas MD Anderson Cancer Center Houston Texas USA

**Keywords:** APG‐157, head and neck cancer, immune checkpoint inhibitors, microbiome, tumor microenvironment

## Abstract

**Background:**

A phase I clinical study for patients with locally advanced H&N cancer with a new class of botanical drug APG‐157 provided hints of potential synergy with immunotherapy. We sought to evaluate the efficacy of the combination of APG‐157 and immune checkpoint inhibitors.

**Methods:**

CCL23, UM‐SCC1 (human), and SCCVII (HPV−), MEER (HPV+) (murine) H&N cancer cell lines were utilized for in vitro and in vivo studies. We measured tumor growth by treating the mice with APG‐157, anti‐PD‐1, and anti‐CTLA‐4 antibody combinations (8 groups). The tumor microenvironments were assessed by multi‐color flow cytometry, immunohistochemistry, and RNA‐seq analysis. Fecal microbiome was analyzed by 16S rRNA sequence.

**Results:**

Among the eight treatment groups, APG‐157 + anti‐CTLA‐4 demonstrated the best tumor growth suppression (*p* = 0.0065 compared to the control), followed by anti‐PD‐1 + anti‐CTLA‐4 treatment group (*p* = 0.48 compared to the control). Immunophenotype showed over 30% of CD8+ T cells in APG‐157 + anti‐CTLA‐4 group compared to 4%–5% of CD8+ T cells for the control group. Differential gene expression analysis revealed that APG‐157 + anti‐CTLA‐4 group showed an enriched set of genes for inflammatory response and apoptotic signaling pathways. The fecal microbiome analysis showed a substantial difference of lactobacillus genus among groups, highest for APG‐157 + anti‐CTLA‐4 treatment group. We were unable to perform correlative studies for MEER model as there was tumor growth suppression with all treatment conditions, except for the untreated control group.

**Conclusions:**

The results indicate that APG‐157 and immune checkpoint inhibitor combination treatment could potentially lead to improved tumor control.

## 1 INTRODUCTION

Therapeutic potential of flavonoid called curcumin, derived from the plant *Curcuma longa*, has been studied for over 20 years in cancer medicine. It has been shown that curcumin can inhibit cancer cell growth by inhibiting cell cycle progression, and activating apoptosis and autophagy.^1^, ^2^ In clinical studies, curcumin showed inhibitory effect on immune suppressive cytokines.^3^ However, clinical application of curcumin has been hampered due to its poor oral absorption and rapid degradation.^4^

APG‐157, a first‐in‐class botanically derived immune‐oncology drug, consists of curcumin along with other pharmacologically active molecules derived from *C*. longa. It was developed based on one of the principles of evolutionary biology that the combination of multiple molecules resulting from a common biosynthetic pathway during the production of secondary metabolites can be far more efficacious in addressing a biological problem than a single molecule due to natural synergy of action among such molecules. Moreover, APG‐157 was formulated to be delivered in a hydrogel pastille via an oromucosal administration. In H&N cancer, such a delivery allows both topical absorption of the drug and also systemic absorption via buccal absorption. When this formulation was tested in our phase I clinical trial,^5^ it showed no adverse events. The clinical observations further suggested that APG‐157 has a great potential to be combined with immune checkpoint blockade. For example, APG‐157 treatment markedly enhanced CD8+ T‐cell infiltration into the TME and decreased concentrations of T‐cell inhibitory cytokines (IL‐1β, TNF‐α, and IL‐8), in saliva and serum. Interestingly, 16S RNA sequencing of oral microbiome of study subjects showed a reversal from high levels of *Bacteroides* genus and low levels of *Firmicutes* before the drug administration toward a more normal level of Firmicutes to *Bacteroides ratio* after 24 h of APG‐157 treatment in cancer subjects. Although a recent study showed no significant associations between oral bacterial diversity and clinical response to immunotherapy,^6^ that study was limited by the fact that there was no topical administration of the drug such as APG‐157 that is more likely to induce microbial changes. To evaluate potential utility of immunotherapy combination treatment with APG‐157, we performed in vitro and in vivo preclinical studies with APG‐157 using human and murine H&N cancer cell lines and immunocompetent mice.

For in vitro studies, we utilized HPV+ (CCL23) and HPV− (UM‐SCC1) cell lines and assessed the cell proliferation with APG‐157 in the presence or absence of interferons (IFNs) since IFN signaling plays a significant role in mediating response or resistance to checkpoint blockade immunotherapy.^7^, ^8^, ^9^, ^10^ For in vivo studies, we utilized immunocompetent C3H/HeOuJ mice for subcutaneous tumor growth by injecting murine H&N cancer cell lines (SCCVII and MEER) and determined anti‐tumor activities of several combinations of immune checkpoint inhibitors, including anti‐CTLA‐4 antibody and APG‐157. Analysis of gut microbiome of the mice treated with APG‐157 and immune checkpoint inhibitors have shown significant shifts in gut microbial species which might be associated with treatment response.

## 2 RESULTS

### 2.1 Effects of APG‐157 and curcumin with and without interferons in cell proliferation, NF‐κB, apoptosis, and cell cycle

**FIGURE 1:**
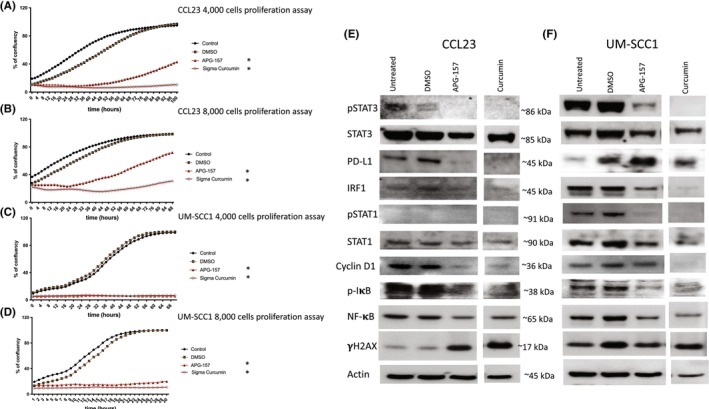
Proliferation and western blot analyses of CCL23 and UM‐SCC1 human H&N cancer cell lines with treatment of APG‐157 and curcumin derivatives. (A, B) CCL23 proliferation assay with curcumin derivatives (8 μg/mL of APG‐157, and Sigma Curcumin) by seeding 4000 and 8000 cells into 96‐well plates. Cell density was measured by live cell imager, incucyte. APG‐157 was compared to curcumin, all of the unpaired t tests reached statistical significance. (C, D) UM‐SCC1 proliferation assay with curcumin derivatives (8 μg/mL of APG‐157, and Sigma Curcumin) by seeding 4000 and 8000 cells into 96‐well plates. Incucyte measured cell densities as stated above. As CCL23, APG‐157 showed stronger inhibitory effect on UM‐SCC1 compared to CCL23. *p < 0.0001 comparing control versus each treatment group (unpaired, nonparametric *t*‐test). (E, F) Western blot analyses of CCL23 and UM‐SCC1 for IFN, NF‐κB pathways and γH2AX. The cells were treated with DMSO and curcumin derivatives for 2 days, cells were collected for cell lysis, and these blots represent at least two independent experiments.

APG‐157 is currently under phase II clinical study as a neoadjuvant treatment for patients with locally advanced H&N cancer. We tested growth inhibitory effects with several concentration to identify suitable concentration to combine with interferon treatment. We performed MTT assays and observed approximate IC50 for APG‐157 is 29.8 μg/mL for CCL23 cells (data not shown). Then, we tested two seeding densities (4000 and 8000 cells per well) using CCL23 and UM‐SCC1 cells by treating them with 5 or 10 μg/mL of APG‐157 and Curcumin. APG‐157 at 10 μg/mL showed a strong growth inhibitory effect for both the seeding densities (data not shown). Then, we re‐assessed the proliferation of these two cell lines with 8 μg/mL of APG‐157 and Curcumin, found growth inhibitory effect (Figure 1A–D). Western blot analysis of IFN signaling, cell cycle, and NF‐κB pathways in CCL23 cells demonstrated relatively inert IFN signaling as opposed to UM‐SCC1 (Figure 1E,F). Notably, PD‐L1 expression was down‐regulated in CCL23, but up‐regulated in UM‐SCC1 by APG‐157.

Despite the fact that interferon (IFN)‐γ can induce growth inhibition in cancer cells through immune‐sensitization, the growth inhibitory effect is not uniform among various cancer cells. Based on unpublished observations, approximately 35%–40% of melanoma cell lines (a total 55 cell lines have been tested) have more than 50% of cell growth inhibition with IFN‐γ. Interestingly, this may be in alignment with the general response rate to immune checkpoint blockade therapy for patients with advanced melanoma. This opens an opportunity to identify potential agents or genes that might work synergistically or additively with IFN‐γ to induce enhanced growth inhibition using the concept of synthetic lethality. Based on our previous experience with IFN‐γ in melanoma model, we performed proliferation assay on CCL23 and UM‐SCC1 cells by treating them with IFNs with and without APG‐157.

The required concentration of APG‐157 for the combination treatment with IFNs was determined by seeding two different cell numbers of UM‐SCC1 cells while the IFN concentrations were based on our previous report (Figure S1A,B).^7^ IFNs alone showed moderate degree of inhibitory effect on proliferation whereas APG‐157 (8 μg/mL) combined with IFN‐γ demonstrated maximum inhibitory effect (Figure S1B). We treated UM‐SCC1 or CCL23 cells with APG‐157 (8 μg/mL) with or without IFNs and the results showed maximum inhibitory when APG‐157 was combined with IFN‐β (Figures S1C–F). We subsequently assessed IFN signaling, apoptosis, and NF‐κB with IFNs at 0, 8, or 20 μg/mL of APG‐157 at three different time points (6, 18, and 48 h) (Figure S1G). The degree of induction of PD‐L1/STAT/p‐STAT1 was higher with IFN‐γ, but p‐STAT3 was substantially lower with IFN‐γ compared to IFN‐β treatment. However, when IFN‐γ was combined with APG‐157, there was robust p‐STAT1 induction and substantial reduction of p‐IκB/NF‐κB compared to IFN‐γ alone, particularly at the 48‐h time point. The in vitro proliferation assays on multiple human (H1975, H1703, A226, A427, RH2, and HCC827) and murine cancer cell lines (SCCVII, MEER+, MC38, and KPL) showed growth inhibitory effect with APG‐157 in the presence of IFNs (Figure S2).

### 2.2 APG‐157 oral administration with food shows absorption of curcumin and components in mouse tissues

We first determined the best mode of providing APG‐157 for tissue absorption using food, drinking water, and i.p. modes of APG‐157 delivery (Figure S3A). We tested four mice each with APG‐157 via food (1% w/w in mouse chows), in water (2.5 mg/mL for 7 days and 10 mg/mL for 3 more weeks, also containing 5% sucrose to compensate for the taste), via i.p. (intraperitoneal injection 5 days a week of 0.5 mg in 200 μL pharmaceutical grade saline/mouse for 1 week and 1.0 mg in 200 μL saline/mouse for 3 more weeks). The control mice received regular mouse chow. Blood was collected through tail vein on a weekly basis and at the end of the 4‐week period when the mice were sacrificed. At the time of sacrifice, we also collected liver, kidney, and spleen tissues. Absorption of the different components of APG‐157 were measured using mass spectrometry. The results showed the presence of curcumin and its derivatives in the serum and liver of APG‐157 food treated group.^11^ The APG‐157 components were not detected in the control, APG‐157 in water and i.p. administration groups (Figure S3B–E). CBC and chemistry studies did not show any toxicity (data not shown), and mice weights from all three groups were comparable to the control group (Figure S3F–J). Thus, the results indicated that APG‐157 (1% w/w in mouse chow) is consumed and the APG‐157 components are absorbed into the circulating blood cells and mouse tissues. It is considered that when a mouse is chewing the APG‐157 containing food, it is absorbed through oral mucosa as we have seen in human trials (where the pastille is kept in oral cavity for slow absorption). Based on these results, we supplied 1% APG‐157 in mouse chow for the subsequent studies.

### 2.3 APG‐157 enhances anti‐tumor activity in combination with anti‐CTLA‐4 antibody

**FIGURE 2:**
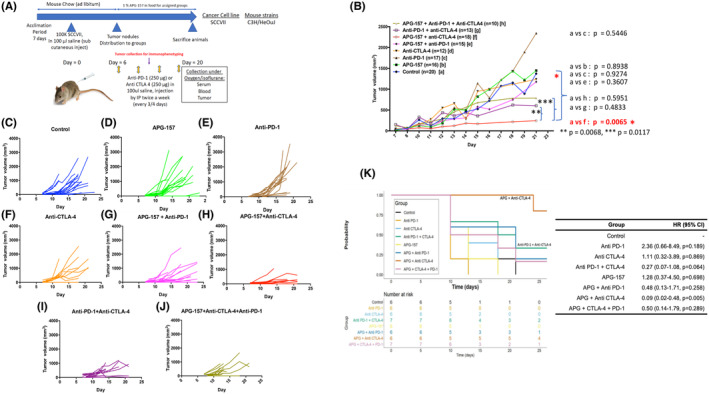
Anti‐tumor activity of APG‐157 and immune checkpoint inhibitors on syngeneic murine SCCVII H&N cancer model. (A) Work flow of mice experiments to assess anti‐tumor activity of APG‐157 and immune checkpoint inhibitors. C3H/HeOuJ mice were purchased from Jackson laboratory, and 100,000 SCCVII cells were injected into flank after 7 days of acclimation period. Once the palpable tumor nodules were confirmed (50–100 mm^3^), mice were randomized to each group and initiated treatment, total 8 groups including control (saline injection). 250 μg of Anti‐PD‐1/CLTA‐4 antibodies was injected 4 times with 3–4 days interval while APG‐157 were administered with food continuously until the end of experiments. Upon sacrifice, tumor and spleen were collected. For immunophenotyping of the tumor, selected mice were sacrificed between Days 12–13 (after the two injections of immune checkpoint inhibitors). (B) Anti‐tumor activity measured by tumor volume (mm^3^) for each treatment group. Control group was compared to each treatment group, APG‐157 + Anti‐CTLA‐4 treatment demonstrated statistically significant tumor control. APG‐157 + Anti‐CTLA‐4 group showed statistically significant tumor control compared to Anti‐PD‐1/CTLA‐4 and APG‐157 + Anti‐PD‐1/CTLA‐4 groups (Mann–Whitney test, *p*‐value stated in the figure). (C–J) Individual mice tumor volume changes in each treatment group. (K) Kaplan–Meier survival curves. The data were submitted to a Cox proportional hazards regression analysis. Compared to the control group, mice in the APG + Anti‐CTLA‐4 group had a significantly reduced risk of death (HR = 0.09, 95% CI [0.02, 0.48], *p* = 0.005). The reduction in risk of death for the Anti‐PD‐1 + CTLA‐4 group was marginally significant (*p* = 0.064).

We optimized the number of murine H&N cancer cells, SCCVII using C3H/HeOuJ mice, and determined that 100,000 cells would yield subcutaneous tumor when injected into the mice flanks (Figure 2A). After the cell injection, the mice were monitored until the tumor reached palpable size, and the mice were then assigned to different groups. Mouse anti‐PD‐1 and anti‐CTLA‐4 antibodies were administered every 3–4 days via intraperitoneal injection for a total of 4 times. Around Day 12, we collected tumors for immunohistochemistry and immunophenotyping by flow cytometry. Mice were sacrificed on Day 21, and tumors were collected. Tumor growth analysis showed that APG‐157 combined with the anti‐CTLA‐4 antibody suppressed tumor growth (Figure 2A,B). Individual group tumor growth data pointed to higher growth of anti‐PD‐1 treated mice (Figure 2C–J). Survival analysis by Kaplan–Meier and Cox Proportional Hazard model showed that APG‐157 + anti‐CTLA‐4 group had the best survival suggesting that this combination did not result in any toxicity (Figure 2K).

### 2.4 APG‐157 and immune checkpoint inhibitor combination treatments recruit T cells into tumor microenvironment (TME)

**FIGURE 3:**
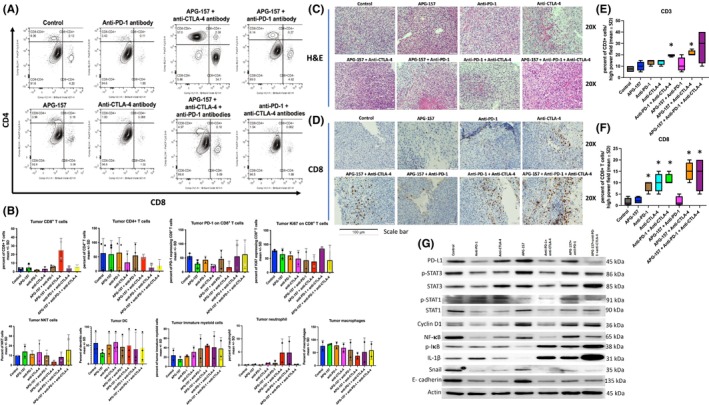
Increased CD8+ T‐cell infiltration with APG‐157 + Anti‐CTLA‐4 treatment. (A) Each tumor sample was subject to immunophenotyping via flow cytometry by staining with CD8 and CD4 antibodies. APG‐157 and Anti‐CTLA‐4 treatment groups showed markedly increased CD8+ T cells compared to other groups. (B) Percent of each subtype of immune cells by immunophenotyping of the tumors measured by flow cytometry. At least two data points were used for this analysis. CD8 T cells were enriched in the APG‐157 + Anti‐CTLA‐4 group; however, it was statistically not significant, most likely due to the limited number of data points. The rest of subset analysis did not show statistically significant changes. (C) Hematoxylin and eosin staining of the mice tumors for each group. (D) Immunohistochemical staining with anti‐CD8 antibody. (E, F) Quantitative analysis of CD3 and CD8+ T cells from each treatment group, respectively. *p < 0.05. Each data point derived from percentage of CD3 and CD8 positive cells per high power fields (×40). (G) Western blot analysis of the tumors from each treatment group. APG‐157 + Anti‐CTLA‐4 group is missing due to insufficient tumor (available tumor sample was used for flow cytometry). The western blot analysis of this group is shown in Figure 5P along with CD8+ T‐cell depleted groups. Immunoblot was performed for IFN signaling molecules (PD‐L1, STAT3, STAT1), NF‐κB/p‐IκB and epithelial‐mesenchymal transition (EMT) markers (Snail, E‐cadherin, and IL‐1β). This represents two independent experiments.

We observed marked increase in CD8+ and CD4+ T‐cell population in post APG‐157 tumor biopsy in a recent phase I clinical trial.^5^ In the present study, we assessed TME by flow cytometry for SCCVII tumors. There was a 10‐fold increase in the proportion of CD8+ T cells (35% vs. 4%) in the APG‐157 and anti‐CTLA‐4 antibody treated group in comparison with the untreated control group (Figure 3A). Due to limited tumor samples, the CD8+ T‐cell assessment by flow cytometry did not reach statistical significance (*p* = 0.2, for Control vs. APG‐157 + anti‐CTLA‐4, Figure 3B). Most of the other groups showed a proportion of 2%–5% of CD8+ T‐cell population. For the CD4+ T cells, we observed variabilities and were not statistically significant (data not shown). We also assessed the values of subset of immune cells using the flow cytometry (Figures S4 and S5). Although statistically not significant, there was a trend toward increase in the population of tumor neutrophil and decrease in NKT population for APG‐157 and anti‐CTLA‐4 combination (Figure 3B). Interestingly, multiple repeat experiments showed accelerated tumor growth with anti‐PD‐1 single therapy group.

We assessed the tumor histology by H&E and immunohistochemistry by staining for the expression of CD3 and CD8; there was a significant increase in CD3+ T cells in Anti‐PD‐1/CTLA‐4 and APG‐157/Anti‐CTLA‐4 groups (Figure 3C–F). CD8+ T cells were significantly increased for most of the groups except for APG‐157 and APG‐157/Anti‐PD‐1 groups (Figure 3F). There was significant increase in PD‐1 expression for anti‐PD‐1 and anti‐CTLA‐4 treated group; Foxp3 expression was increased for triple combination group (APG‐157 + anti‐PD‐1 + anti‐CTLA‐4) (Figure S6A–H). Western blot analyses were performed for all the groups except for APG157 + anti‐CTLA‐4 groups because there was no tumor available for this analysis. However, one small‐resected tumor was available from this group for multi‐color flow cytometric analysis and immunohistochemistry presented above. APG‐157 treatment suppressed NF‐κB and p‐IκB expression whereas single immunotherapy groups suppressed NF‐κB, but not p‐IκB (Figure 3G). A possible reason for the single immunotherapy group showing inhibition of NF‐κB, but not p‐IκB could be due to inhibition of noncanonical NF‐κB pathway or AKT pathway which are not targeted by APG‐157.^12^ The APG‐157 + immunotherapy combination groups, however, showed down‐regulation of NF‐κB and increased expression of p‐IκB, possibly again through a noncanonical NF‐kB signaling pathway. APG‐157 treatment in general did not affect IFN signaling molecules (STAT1, pSTAT1, STAT3, and pSTAT3), although PD‐L1 expression appears to show a slight increase in comparison with the untreated control. Single checkpoint inhibitor therapies or combined with APG‐157 or double immunotherapy decreased p‐STAT1 and PD‐L1 expression (in these blots, resected samples represent both the tumor, stromal and immune cells in the TME).

We also assessed IL‐1β and epithelial to mesenchymal transition (EMT) markers (snail and E‐cadherin) with tumors treated with APG‐157 and immune checkpoint inhibitors. The role of IL‐1β in modulation of EMT markers (E‐cadherin and snail) has been studied earlier, indicating that IL‐1β enhanced snail expression led to increased metastatic burden in the H&N cancer cell line model.^13^ Interestingly, APG‐157 inhibited IL‐1β and snail, while slightly increasing the expression of E‐cadherin. Single checkpoint inhibitor therapies or combination groups demonstrated suppression of snail and E‐cadherin suggesting that there might be involvement of other EMT markers (examples include Twist and Zeb1) in the SCCVII tumor cell growth (Figures 3G and 5P). IL‐1β expression was significantly increased among combination groups although it was almost completely lost with APG‐157 treatment. This suggests that IL‐1β might be increased through noncanonical NF‐κB or other signaling pathways in checkpoint blockade treated groups. Our data also suggest that targeting IL‐1β might improve the efficacy of combination therapies. Western blot analyses of tumors including those from CD8+ T‐cell depletion studies showed down‐regulation of STAT3/pSTAT3, but up‐regulated PD‐L1/STAT1/pSTAT1 in the APG‐157 + anti‐CTLA‐4 antibody group (Figure 5P). NF‐κB was modestly down‐regulated compared to the control; however, there was a substantial difference in NF‐κB down‐regulation compared to anti‐CD8 antibody and APG‐157 + anti‐CTLA‐4/CD8 antibody groups.

We tested another H&N cancer model, MEER (HPV−). The tumor growth experiment was performed with optimization of cell number as Figure S7A shows. The tumor grew substantially slower than SCCVII model, and the therapeutic effect of single CTLA‐4 or anti‐PD‐1 antibodies was substantial. Hence, we were not able to collect tissue samples to perform TME studies with these experiments (Figure S7B–L).

**FIGURE 4:**
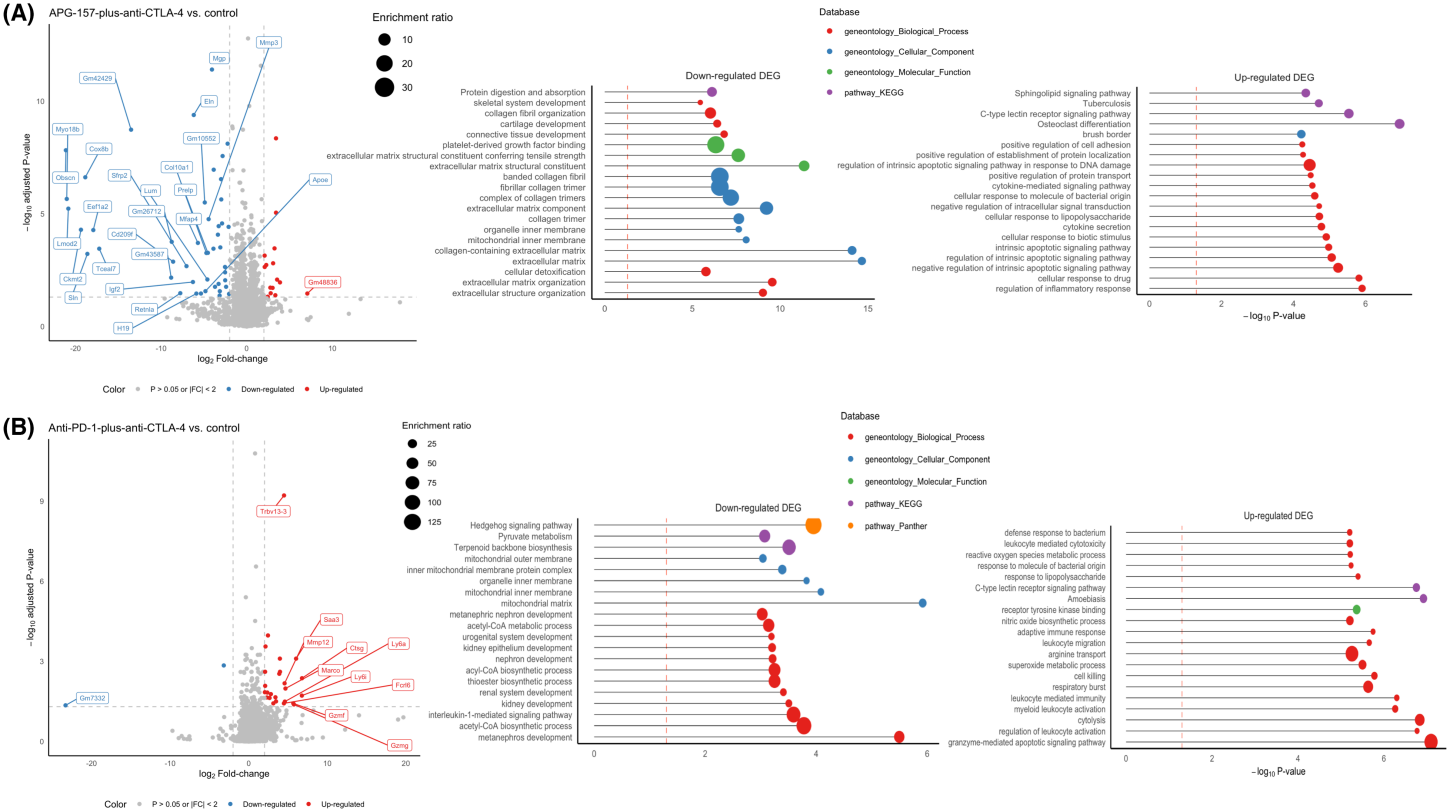
Differential gene expression of the tumors treated with APG‐157 and immune checkpoint inhibitors using RNA‐seq. (A) Volcano plot of the differential gene expression analysis for APG‐157 + anti‐CTLA‐4 versus Control. Significantly down‐regulated genes are annotated by blue colors, and up‐regulated genes are annotated by red colors. Gene ontology (Gene‐Set Enrichment) analysis shows a substantial number of genes and pathways are down‐regulated for APG‐157 + Anti‐CTLA‐4 groups. Banded collagen fibril, complex of collagen trimmers, and extracellular matrix component gene ontology groups were significantly down‐regulated (high enrichment ratio). Among up‐regulated genes, various inflammatory signaling, DNA damage response, and apoptotic signaling pathway gene ontology groups were significantly up‐regulated. (B) Volcano plot of differential gene expression analysis for Anti‐PD‐1 + Anti‐CTLA‐4 versus Control. It shows a minimal number of genes that are significantly down‐regulated contrasted to the number of genes that were significantly up‐regulated which are mostly immune related genes, and this is reflected well by gene ontology analysis. (A, B) The threshold for significance is set at >2 (log2 differential value, *x*‐axis), and *y*‐axis shows statistical value by log_10_ scale.

Differential gene expression from these treatment groups was assessed by RNA‐seq using the tumor samples. Volcano plots and gene ontology (GO) analyses showed substantial differences in up‐regulated and down‐regulated genes (Figure 4A,B; Figures S8 and S9). For control versus APG‐157 + anti‐CTLA‐4, Figure S10 shows the top 10 genes that are significantly down‐regulated. We assessed each of these genes via human protein atlas (TCGA expression data sets) for potential prognostic role in H&N cancer. Interestingly, high expressions of Myo18b, Ckmt2, and Eef1a2 showed unfavorable prognoses in H&N cancer, and Sln (sarcolipin) and Tceal7 are unfavorable for renal cell carcinoma. High Obscn (top of the down‐regulated genes) expression showed a trend toward unfavorable prognosis in H&N cancer (Figure S10). Thus, down‐regulation of genes related to poor prognosis correlated well with improved tumor control seen in APG‐157 + anti‐CTLA‐4 antibody group. Among the top 10 up‐regulated genes shown in Figure S11, several are related to T‐cell cytotoxic activity, such as Gzma, Gzmb, and Prf1.^14^ In addition, increased expression of TCR‐β chain variable 13‐3 might (Trbv13‐3) suggest expansion of a specific set of CD8+ T cells targeting tumor antigens. Among other top 10 up‐regulated genes, Scl7a2 suggestive of inflamed gene signature for H&N cancer and potentially a better prognostic marker.^15^ Another up‐regulated gene Acod1 has been shown to play a role in tumor associated macrophage (TAM) metabolic reprogramming.^16^ Pires‐Afonso et al. have shown that decreased antigen presenting cell features and immune reactivity in TAMs during tumor progression are enhanced in Acod1‐deficient mice. For Mmp12 and Ltb4r1 genes, the high Mmp12 expression shows a trend toward better prognosis in H&N cancer (Human Protein Atlas). The role of Ltb4r1 is still not clearly understood (Figure S11). When the anti‐PD‐1 + anti‐CTLA‐4 group is compared with the control we have observed that most of the differentially expressed genes that are up‐regulated (cut off by 2‐fold change with log_2_ scale) are related to a subset of immune cell function (Figure 4B). Interestingly, the TCR‐β chain variable 13‐3 is also up‐regulated in this analysis, which we think further supports our hypothesis that T cells with this variable β chain might target tumor antigens.

### 2.5 Depletion of CD8+ T cell abolishes anti‐tumor activity of APG‐157 and anti‐CTLA‐4 combination treatment

**FIGURE 5:**
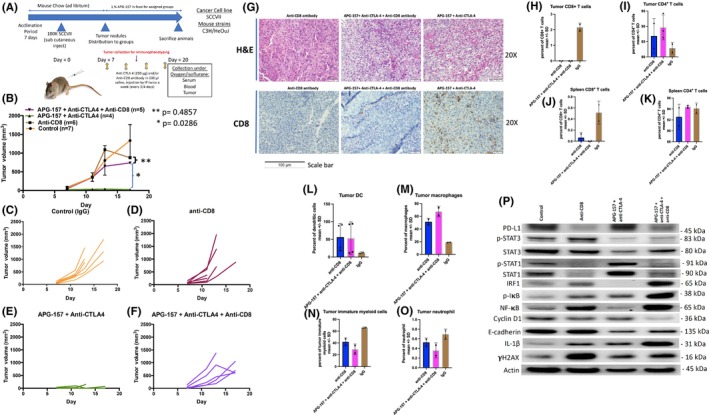
Depletion of CD8+ T cell abolished the anti‐tumor activity of APG‐157 and anti‐CTLA‐4 antibody treatment. (A) Work flow of mice experiments by depleting C8+ T cell by anti‐CD8 antibody. After the acclimation period, mice were subject to 100,000 SCCVII cancer cells into flanks, when the tumors reach to palpable size as described in Figure 2, mice were randomized into four groups, (i) Control, (ii) Anti‐CD8 antibody, (iii) APG‐157 + Anti‐CTLA‐4, and (iv) Anti‐CD8 antibody + APG‐157 + Anti‐CTLA‐4. APG‐157 was administered with food and Anti‐CD8, anti‐CTLA‐4 antibody were administered by intraperitoneally. Tumors were collected for immunophenotyping at Days 11–13 and sacrificed at Days 20–23. (B) Tumor volumes were plotted in the panel, and anti‐CD8 antibody + APG‐157 + Anti‐CTLA‐4 showed no difference compared to control or anti‐CD8 antibody groups. APG‐157 + Anti‐CTLA‐4 versus anti‐CD8 + APG‐157 + Anti‐CTLA‐4 was significant; *p* values were noted in the panel (Mann–Whitney test). (C–F) Individual mice tumor volumes were plotted for each group. (G) Upper panel: Hematoxylin and eosin, lower panel: CD8 IHC of the tumors. Image was taken at 20×, scale bar represents 100 μM. (H–K) percent of CD8+/CD4+ T cells of the tumor and spleen assessed by flow cytometry. (L–O) percent of dendritic cell, macrophage, immature myeloid cells, and neutrophil of the tumors assessed by flow cytometry. (P) Western blot analyses of the four groups of treatment. Immunoblots were performed for IFN (PD‐L1, STAT1, STAT3, IRF1), NF‐κB/p‐IκB, EMT markers (E‐cadherin, and IL‐1β), and autophagy markers (ATG7, LC3B, and Beclin). The blot represents at least two experiments.

To assess the role of CD8+ cytotoxic T cells in the anti‐tumor activity of the combination treatment with APG‐157 and anti‐CTLA‐4 antibody, we performed depletion studies using anti‐CD8 antibody as shown in Figure 5A. The SCCVII subcutaneous tumors were treated with IgG or anti‐CD8 or APG‐157 + anti‐CTLA‐4 or APG‐157 + anti‐CTLA‐4/CD8 antibodies (Figure 5A). Control, anti‐CD8, and APG‐157 + anti‐CTLA‐4 + anti‐CD8 groups had tumor cell growth demonstrating loss of anti‐tumor activity when CD8+ T cells were depleted for the APG‐157 + anti‐CTLA‐4 treatment group (Figure 5B). Tumor growth of individual treatment groups indicated that there was no significant differential tumor growth among IgG, anti‐CD8, and APG‐157 + anti‐CTLA‐4 + anti‐CD8 antibodies treated groups (Figure 5C–F). Again, APG‐157 + anti‐CTLA‐4 treatment group demonstrated tumor growth suppression. The CD8 depletion by anti‐CD8 antibody was effective as we do not see CD8+ T cells in the treatment groups (Figure 5G). Immunophenotype by multi‐color flow cytometry for the tumor and spleen showed that other than CD8+ T‐cell changes, there was no significant changes for CD4+ T cell, tumor DC (dendritic cell), tumor macrophage, tumor immature myeloid cells, and tumor neutrophils (Figure 5H–O). Western blot analyses of the tumors showed up‐regulations of PD‐L1, STAT1, p‐STAT1, and IRF‐1 expression in a single small tumor from APG‐157 + anti‐CTLA‐4 treated group (Figure 5P). There was also significant down‐regulation for STAT3, p‐STAT3, and NF‐κB. The expression patterns seen with APG‐157 + anti‐CTLA‐4 treatment were reversed post CD8+ T‐cell depletion (see Figures 3G and 5P).

### 2.6 APG‐157 shifts microbial species in mice with checkpoint inhibitor combination treatment

**FIGURE 6:**
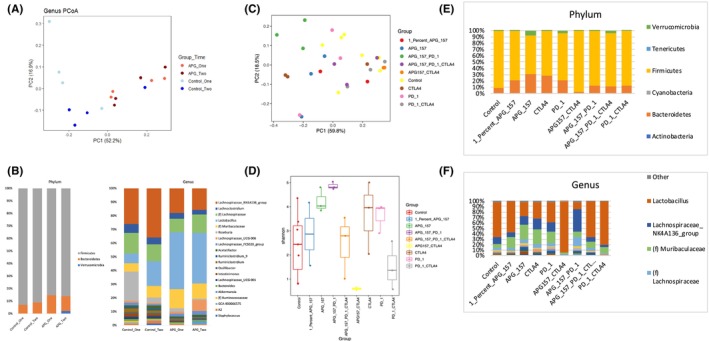
APG‐157 and Anti‐CTLA‐4 treatment significantly increased *Lactobacillus* microbial species in mice bearing SCCVII tumor. (A) Principal component analysis of 16S sequencing of the fecal material from the control and APG‐157 treated groups. The stool samples were collected prior to APG‐157 administration and after 4 weeks of administration. (B) Phylum and genus analysis of the 16S sequencing of the fecal material from panel A. After 4 weeks of APG‐157 administration, *Lactobacillus* population increased significantly. (C) Principal component analysis of 16S sequencing from each treatment group (APG‐157 and immune checkpoint inhibitors). (D) Shannon diversity analysis of each group using 16S sequencing. APG‐157 + Anti‐CTLA4 group showed the lowest diversity score among the group. (E, F) Phylum and genus analysis. APG‐157 + Anti‐CTLA‐4 group demonstrated increased Firmicutes and Genus analysis revealed that this treatment group has significant increase in the population of *Lactobacillus*.

We sought to assess whether APG‐157 would change the composition of the gut microbiome in mice. We utilized C3H/HeOuJ mice and treated them with APG‐157 and all other treatment groups as discussed earlier (Figure 6). Principal component analysis of the fecal microbiome treated with two different concentrations of APG‐157 (one = 1% of APG‐157, two = 2% of APG‐167) showed distinct differences in microbial species in comparison with untreated controls (Figure 6A). The phylum and genus analyses showed a significant increase of *Lactobacillus* species (Figure 6B). Fecal microbiome principal component analysis of the treatment groups revealed dispersed microbial groupings (Figure 6C). Microbial diversity assessed by Shannon analysis showed APG‐157 + anti‐CTLA‐4 antibody treatment group to have the lowest diversity score (Figure 6D). Phylum and genus analyses, respectively, indicated that APG‐157 + anti‐CTLA‐4 antibody treatment group shifted microbiome mostly to Firmicutes (phylum analysis) and *Lactobacillus* (Genus analysis) (Figure 6E,F).

## 3 DISCUSSION

Natural compounds found in plants have been the source of drug discovery over the past several decades, not only for cancer treatment but also for other human diseases.^17^ As we understand more of the biological activities and mechanisms of action for these compounds from the preclinical and clinical studies, we will gain insight into their utility in cancer therapy.^18^ Curcumin is one such natural compound that has been studied extensively over decades and clinical studies have been performed to test its utility as a cancer treatment.^19^ While many preclinical and some clinical studies were encouraging, its full clinical efficacy has not been realized. The overall approach to developing the drug based on curcumin has followed the reductionistic paradigm of a single agent synthetic drug which fails to leverage the evolutionary advantages of a natural, combination therapeutic where curcumin's pharmacological activity can be substantially enhanced. Superior pharmacological performance can result from synergistic interaction of multiple molecules—derived from a common biosynthetic pathway along with curcumin—where each molecule is designed to play a distinct role ranging from its versatility in binding to the proteins of interest, to avoidance of the toxicity, to enabling the absorption of the pharmacologically active molecules.

Therefore, APG‐157 was rationally designed by combining curcumin, the principal molecule, with other co‐evolved molecules from *C. longa*. Curcumin has been reported to suffer from efficient first‐pass metabolism and particularly glucuronidation and sulfation of curcumin, might explain its poor systemic availability when administered via the oral route and lackluster clinical effect in some cases.^20^ APG‐157 was developed to address the issue and enhance the systemic and local absorption. The drug substance containing curcumin was formulated as an oral hydrogel pastille not only to enhance systemic absorption but also enhance local direct effect on cancer cells in oral cavity.^5^ In Phase 1 study, subjects had no adverse events and correlative study has revealed anti‐inflammatory properties (decrease in inflammatory cytokines and enhanced T‐cell infiltration into TME) that could be exploited for the combination treatment with immunotherapy. Curcumin is reported to modulate the proliferation and activation of T cells. It can either inhibit or induce the T‐cell proliferation depending on its dose administered.^21^ Currently, APG‐157 is studied for patients with locally advanced H&N cancer as a neoadjuvant treatment for 4 weeks prior to surgical or chemo‐radiation treatment at our institution and so far, results have been encouraging (NCT053212710).

Cancer immunotherapy using immune checkpoint inhibitors has been a breakthrough development for patients with many types of cancer including H&N cancer. Many studies have demonstrated that tumor interferon signaling plays a critical role in mediating response and resistance.^7^, ^8^, ^9^, ^10^ Precision oncology or personalized medicine has become a reality with molecularly targeted therapies for patients with various types of malignancies. However, the major challenge with these targeted therapies is that majority of patients will eventually develop acquired resistance after initial response reflecting the cancer cell's remarkable adaptability or plasticity. Therefore, one might consider a drug that inhibits multiple signaling pathways or genes would prevent or delay the development of resistance. The field of targeted therapy has been testing various combinations to address drug resistance, and a great example is BRAF targeted therapy for patients with metastatic melanoma.^22^ However, these combination treatments are often challenging to administer due to significantly increased toxicities.^23^ In contrast, APG‐157 possesses the properties of inhibiting multiple oncogenic signaling pathways, including NF‐κB, and activate cell cycle senescence and at the same time have reduced toxicities. When this multiple signal targeting APG‐157 is combined with immunotherapy in vivo or IFNs in vitro, we have observed significant improvement in anti‐tumor activity and in cell proliferation inhibitory effect, respectively. Moreover, we have not observed toxicity that could limit the use of combination treatment in the in vivo studies. In addition, we have observed that APG‐157 shows complete tumor cell growth suppression in SCCVII murine H&N cancer model when it is combined with anti‐CTLA‐4 antibody which is dependent on CD8+ T cells in the TME (Figure 5B). This preclinical study is predominantly performed with mice as it potentially reflects human's biological or immunological response. Yet, we also understand the clear difference, particularly the immune system of mice versus human.^24^ Thus, we need to consider the immune differences as we interpret our results of the mice study. It appears that mice with some cancer models may have better response with anti‐CTLA‐4 antibody than anti‐PD‐1 antibody contrary to the observation in human cancers.^25^ SCCVII cell line model was tested by Sato‐Kaneko et al., which showed significant tumor control with anti‐PD‐1 antibody.^26^ This may indicate that clonal variant may influence the responsiveness to immune checkpoint inhibitors. Hence, we believe that the results presented here may provide the rationale to test the combination treatment of APG‐157 with immune checkpoint inhibitors, including anti‐PD‐1 antibody as it is the current backbone of majority of clinical studies in the combinatorial immunotherapeutic investigations.^27^

The role of microbiome in tumorigenesis and treatment response particularly for immunotherapy has been an intense research focus over the past 5–7 years. Various studies have suggested that there is an association between particular microbiome and immunotherapy response or resistance.^28^ Recent study by Owens et al. has suggested that *Lactobacillus rhamnosus GG* augments immunotherapy efficacy for colorectal cancer.^29^ Not only the treatment efficacy, various studies have suggested that *Lactobacillus* has immunomodulatory properties potentially alleviating treatment side effects (particularly for diarrhea) and general inflammatory response related to other diseases.^30^ Most recent report by Bender et al provided mechanistic insight of how bacterial translocation into TME can impact anti‐tumor activity by aryl hydrocarbon receptor signaling in T cells.^31^ Our results on gut microbial shift studies of APG‐157 + anti‐CTLA‐4 antibody treatment indicated that there is enhanced anti‐tumor immune response of the SCCVII H&N cancer cells possibly partly due to *Lactobacillus*.

In summary, we have observed the best tumor control for SCCVII model in immunocompetent mice studies with APG‐157 and anti‐CTLA‐4 combination treatment. The marked increase in adaptive immune cell infiltration into TME with this treatment is potentially mediated by gut microbial shift and multi‐oncogenic signaling inhibition. As we see encouraging results from ongoing phase II neoadjuvant study with AGP‐157, our study result warrants further trials of immunotherapy combination treatment for patients with locally advanced or advanced H&N cancer.

## 4 MATERIALS AND METHODS

### 4.1 Cell Lines

Human H&N cancer cell lines (UM‐SCC1 and CCL23) murine H&N cancer cell (SCCVII and MEER (HPV+)) lines were cultured with complete media (RPMI 1640) containing 10% fetal bovine serum, penicillin (100 U/mL), streptomycin (100 μg/mL), and amphotericin B (0.25 μg/mL). SCCVII cell line was a generous gift from Dr. Grandis at University of California, San Francisco and MEER cell line is from Dr. Cohen at University of California, San Diego. Cell lines were confirmed mycoplasma negative using mycoplasma detection kit (Biotool #B3903) and periodically tested for authentication.

### 4.2 Animals

In the syngeneic mouse model system, C3H/He‐OuJ and C57/BL6 mice are the immunocompetent animal species for syngeneic tumor formation using the SCCVII and MEER cell lines, respectively. Subcutaneous injections will be performed into immune competent C3H/He‐OuJ or C57/BL6 mice for the SCCVII or MEER cell lines. We will inject 100,000 (SCCVII) or 2 million (MEER) cells in 200 μL volume of pharmaceutical grade saline solution will be injected. Animals are housed in animal facility room in bldg. 113. The two mouse species are obtained from the Jackson Laboratory, 600 Main Street, Bar Harbor, Maine. Animals are housed in sterile rodent microisolator caging, with filtered cage top. Two to four animals will be housed in each cage, with animals resting directly on bedding. They are given free access to sterile water and food. All cages, covers, and bedding are sterilized weekly. Tumor volume is calculated using the formula *V* = 4/3*πW*2*L* where *W* is half of shorter axis diameter and *L* is half of longer axis diameter as described. Tumors were excised when the volume reached a maximum of 15 mm in one of the dimensions. All animal procedures were approved by the Institutional Animal Care and Use Committee of the West Los Angeles Veterans Affairs Medical Center, in accordance with the USPHS Policy on Humane Care and Use of Laboratory Animals. We used InVivoMab anti‐mouse PD‐1 (Ref# BE0146) and anti‐mouse CTLA‐4 (Ref# BE0131) or their corresponding isotypes were administered by intraperitoneal injection (250 μg/antibody/dose). The administering schedule is depicted in figures. Survival was monitored and tumors were measured daily until the animals died or the tumor volume reached 1500 mm^3^.

### 4.3 Tumor cell proliferation assay with APG‐157 and interferons

Actively growing CCL23 and UM‐SCCVII cells were seeded at 4000 and 8000 cells/well of 96‐well plate for incucyte experiments and at 10,000 cells/well for MTT assays. Cells were incubated overnight at 37°C with 5% CO_2_, and next day, they were treated with DMSO, Curcumin, and APG‐157 at 5,10,30, 50, 70 and 100 ug/ml for MTT assays. For incucyte experiments, cells were treated with APG‐157 at 1, 4, 8, 16, 18, and 20 μg/mL to determine the optimal cytotoxic concentration. For combinatorial treatment experiments with IFNs, only APG‐157 was used in combination with IFN for treating the cells. Plates containing control and treated cells were then placed in an incucyte, and cell proliferations were monitored for up to 7 days. In parallel experiments, cell viability was monitored by MTT assay following an established protocol. Growth curves generated from the incucyte data are represented the graphs. Similarly, cell viability data obtained by MTT assays were plotted to compare the effects of different treatments.

### 4.4 Immunophenotyping

Characterization of immune cell population was performed using mouse tumor samples which were collected at day 11‐13. Tumor samples were processed to generate single cell suspension using tumor dissociation kit (Miltenyi Biotec, catalog no. 130‐096‐730) per manufacturer's protocol. The samples were stained using two antibody panels, T (T cell) and D (Dendritic and myeloid) panels. T panel includes, PD‐1 (BV785, Biolegened: clone 29F.1A12), CD45 (BV650, Biolegend: clone 30‐F11), CD3(BV605, BD: clone 17‐A2), CD8A (BV421, Biolegend: clone 53‐6.7), Ki67 (FITC, eBioscience: clone SolA15), CD4 (PeCy5, Biolegend: clone RM4‐5), CD25 (APC, eBioscience: clone PC61.5), NKp46 (Ax700, BD: clone 29A1.4), FoxP3 (PE, eBioscience: clone FJK‐16s). D panel includes CD11c (BV650, Biolegend: clone N418), F4/80 (BV421, eBioscence: clone BM8), Ly6C (FITC, BD: clone AL‐21), Ly6G (PerCp5, eBioscience: clone RB6‐8C5), CD11b (APC, eBioscience: clone M1/70), MHC class II (Ax700, eBioscience: clone M5), and PD‐L1 (PE, BD: clone MIH5). Zombie aqua (BV510, Biolegend) was used to discriminate live/dead cell. Cells were stained with surface markers first, then fixed/permeated to stain FoxP3 and Ki67. Cells were analyzed by flow cytometry using a ATTUNE (ThermoFisher) at the UCLA Flow cytometry Core facility. Data were anlayzed using the FlowJo software (version 10.6.1).

### 4.5 Tumor RNA‐Seq analysis

RNA‐seq raw reads, stored as fastq files, were first quality controlled using FastQC 0.12.0 (https://www.bioinformatics.babraham.ac.uk/projects/fastqc/) and trimmed to minimum leading and trailing base qualities of >20, retaining at least 80% of each raw read using Trimmomatic v0.39.^32^ Next, using Salmon v1.5.1^33^ and GENCODE mouse transcriptomic annotations vM27,^34^ transcript‐level quantifications were estimated using a variational Bayes algorithm. Transcript‐level quantifications were imported into R/4.1.0 and summarized to the gene‐level using the tximeta Bioconductor package.^35^ Differential expression between experimental groups and the control groups was assessed using moderated shrinkage estimation of fold changes and dispersion using DESeq2 and apeglm.^36^, ^37^ Overrepresentation analysis of differentially expressed genes was conducted using WebgestaltR.^38^ Lastly, individual cell‐type proportions were estimated from the bulk gene expression and refernce immune cell gene expression profiles from ImmuCC^39^; cell‐type deconvolution was conducted using non‐negative least squares regression in a variance stabilized space using the unmix function from DESeq2.^36^

### 4.6 Immunohistochemistry

Mice tumor samples were fixed with 4% paraformaldehyde; tissue block and slides were processed in TPCL (UCLA pathology service); immunohistochemical staining was performed with validated antibodies. Automated detection was performed using Leica Bond RX processor with the following steps based on the Protocol using Bond Polymer Refine Detection kit (Leica Biosystems, Cat#: DS9800). Heat Induced Antigen Retrieval using ER2 (BOND Epitope Retrieval Solution 2, Leica Biosystems, Cat#: AR9640) buffer at 100°C for 20 min. Peroxide Block 5 min and wash with bond wash buffer three times. Then, stain with primary antibodies (CD8, DAKO/Agilent, Cat#: M7103, 1‐100; CD3 DAKO/Agilent, Cat#: A0542, 1‐100; Ki67, DAKO/Agilent, Cat#: M7240, 1‐100; PDL1, Abcam, Cat#: ab228462, 1‐100; PD1, Cell Marque, Cat#: 315R, 1‐50. CD4, Cell Marque, Cat#: 102R, 1‐50; Foxp3, Ebiosience, Cat#: 14‐4776‐82, 1‐1000) and incubate for 60 min. Then, wash with Bond Wash buffer three times, incubate with post‐primary and HRP polymer from the Bond Polymer Refine Detection kit (Leica Biosystems, Cat#: DS9800) for 10 min. After washing with Bond Wash buffer and deionized water, incubate with Mixed DAB Refine detection system for 10 min. Wash with deionized water and Bond Wash buffer, slides are dehydrated in series of 60%, 70%, and 100% alcohols, cleared with histoclear and mounted with Permount. Slides are read by a pathologist who is blinded for sample identities.

### 4.7 Microbiome analysis

Fecal pellets were collected and stored at −80°C. Using the ZymoBIOMICS DNA Microprep Kit (Zymo Research, USA), DNA extraction was performed according to the kit's guidelines. The V4 segment of the 16S ribosomal RNA gene was subjected to PCR amplification with the 515 F‐806 R primer combination.^40^ These samples were then sequenced using 250 × 2 paired‐end sequencing on an Illumina HiSeq platform (Illumina, San Diego, CA, USA). The generated raw fastq data were refined using the DADA2 workflow in R, wherein taxonomic assignment was based on the SILVA 132 database with standard settings.^41^ After processing in R, the datasets were integrated into QIIME 2 version 2019.10.^42^ To exclude low‐frequency amplicon sequence variants (ASVs), any ASV not detected in at least 15% of the total samples was discarded, a method consistent with our prior works.^43^ The sequencing depths observed spanned from 19,434 to 280,688 for each sample. Using QIIME2, alpha diversity was assessed via the Shannon index, which integrates both species richness and species evenness. For these alpha diversity computations, the dataset was rarefied to a depth of 19,433 reads. Variance analysis (ANOVA) was applied to ascertain the statistical significance of the Shannon index across groups. A post hoc Tukey test was performed to evaluate differences between groups. Beta diversity was ascertained employing the Aitchison distance metric in QIIME2, facilitated by the DEICODE package. Relative to other metrics such as UniFrac or Bray–Curtis, this recent metric more effectively delineates variations.^44^ The differences in beta diversity were analyzed using permutational multivariate analysis of variance via the “adonis” package in R (version 4.1.2).^45^ Differential abundance testing of the different groups versus control was performed using DESEq2 and corrected for multiple hypothesis testing using *q*‐values.

### 4.8 Western blot

Actively growing cells at 70%–80% confluence were treated with APG‐157 and other curcumin derivative compounds at different concentrations for different time periods. After the treatment, cells were lysed in RIPA buffer (Invitrogen, Carlsbad, CA) with added protease and phosphatase inhibitors (Thermofisher Scientific, Waltham, MA). Cell lysates were centrifuged at 15,000 rpm for 10 min, at 4°C and supernatants were collected and proteins were quantified. Western blotting were carried out following established standard protocol. Briefly, appropriate amounts of cell lysates were mixed with 2X loading buffer and heated for 5 min at 90°C. Proteins (40 μg) were run on 4%–12% Bis‐Tris gels (Invitrogen, Carlsbad, CA) and transferred onto PVDF membranes using iBlot 2 (Thermofisher Scientific, Carlsbad, CA). Membranes were blocked with 5% non‐fat dry milk (BioRad, Hercules, CA) in PBST (phosphate‐buffered saline +Tween 20 0.02%) for 1 h, followed by hybridization with the primary antibodies, Actin, NFkB, p‐IKB, Cyclin D1, BCLx/L, BCLx/s, BAX, Cleaved PARP (Santa Cruz Biotechnology, Santa Cruz, CA), PD‐L1, (Novus Biologicals, Centennial, CO), IRF‐1, STAT1, p‐STAT1, STAT3, and p‐STAT3 (Cell Signaling Technology, Danvers, MA) for an overnight at 4°C. Further, blots were washed three times (5 min each time) and incubated with appropriate secondary antibodies, mouse anti‐rabbit IgG‐HRP, or rabbi anti‐mouse IgG HRP (Santa Cruz Biotechnology, Santa Cruz, CA) for 2 h at room temperature. Next, the blots were washed three times as above and developed using chemiluminescence reagents (Supersignal West pico Plus chemiluminescence reagents, Thermofisher Scientific, Carlsbad, CA) and imaged using BioRad Chemidoc imaging system (BioRad, Hercules, CA).

### 4.9 Statistical analysis and reproducibility

Prism 9 software (GraphPad) was utilized to analyze differences between groups and determine statistical significance. For in vitro studies, tumor growth, gene expression, and in vivo tumor growth studies, differences between groups were evaluated using two‐sided unpaired *t* tests. Differences in mouse survival between treatment groups were evaluated using the log‐rank test (cox proportional analysis). Statistical analysis of RNA sequencing data is described above.

## AUTHOR CONTRIBUTIONS

**Daniel Sanghoon Shin:** Conceptualization (lead); data curation (lead); formal analysis (lead); funding acquisition (lead); investigation (lead); methodology (lead); project administration (lead); resources (lead); software (lead); supervision (lead); validation (lead); visualization (lead); writing – original draft (lead); writing – review and editing (lead). **Saroj Basak:** Data curation (equal); formal analysis (equal); investigation (equal); methodology (equal). **Mysore S. Veena:** Data curation (equal); formal analysis (equal); investigation (equal); methodology (equal); writing – review and editing (equal). **Begoña Comin‐Anduix:** Formal analysis (equal); methodology (equal); writing – review and editing (equal). **Arjun Bhattacharya:** Formal analysis (equal); methodology (equal); writing – review and editing (equal). **Tien S. Dong:** Data curation (equal); formal analysis (equal); methodology (equal); writing – review and editing (equal). **Albert Ko:** Data curation (equal); formal analysis (equal). **Philip Han:** Data curation (equal); formal analysis (equal). **Jonathan Jacobs:** Data curation (equal); formal analysis (equal); methodology (equal). **Neda A. Moatamed:** Formal analysis (equal); methodology (equal). **Luis Avila:** Methodology (equal); project administration (equal); writing – review and editing (equal). **Matteo Pellegrini:** Formal analysis (equal); methodology (equal); writing – review and editing (equal). **Marilene Wang:** Project administration (equal); supervision (equal); writing – review and editing (equal). **Eri S. Srivatsan:** Conceptualization (equal); data curation (equal); investigation (equal); methodology (equal); resources (equal); writing – review and editing (equal).

## FUNDING INFORMATION

D.S.S supported by Veterans Affairs Merit award (5I01BX005588‐02) and American Society of Clinical Oncology (ASCO) Career Development Award (CDA, 2018‐2020).

## CONFLICT OF INTEREST STATEMENT

The authors declare no competing interests.

## Supporting information


Data S1.

## Data Availability

APG‐157 Drug Substance PHRAD129 was supplied by Aveta Biomics. Gene expression data will be available at www.ncbi.nih.gov/geo/ (accession number: GSE246084).
