# Supplementary material for: Enhanced CTLA‐4 blockade anti‐tumor immunity with APG‐157 combination in a murine head and neck cancer
Source: Cancer Med. 2024 Apr 30;13(9):e7212. doi: 10.1002/cam4.7212 (PMC11058674; doi:10.1002/cam4.7212)

Supplemental Figure S1

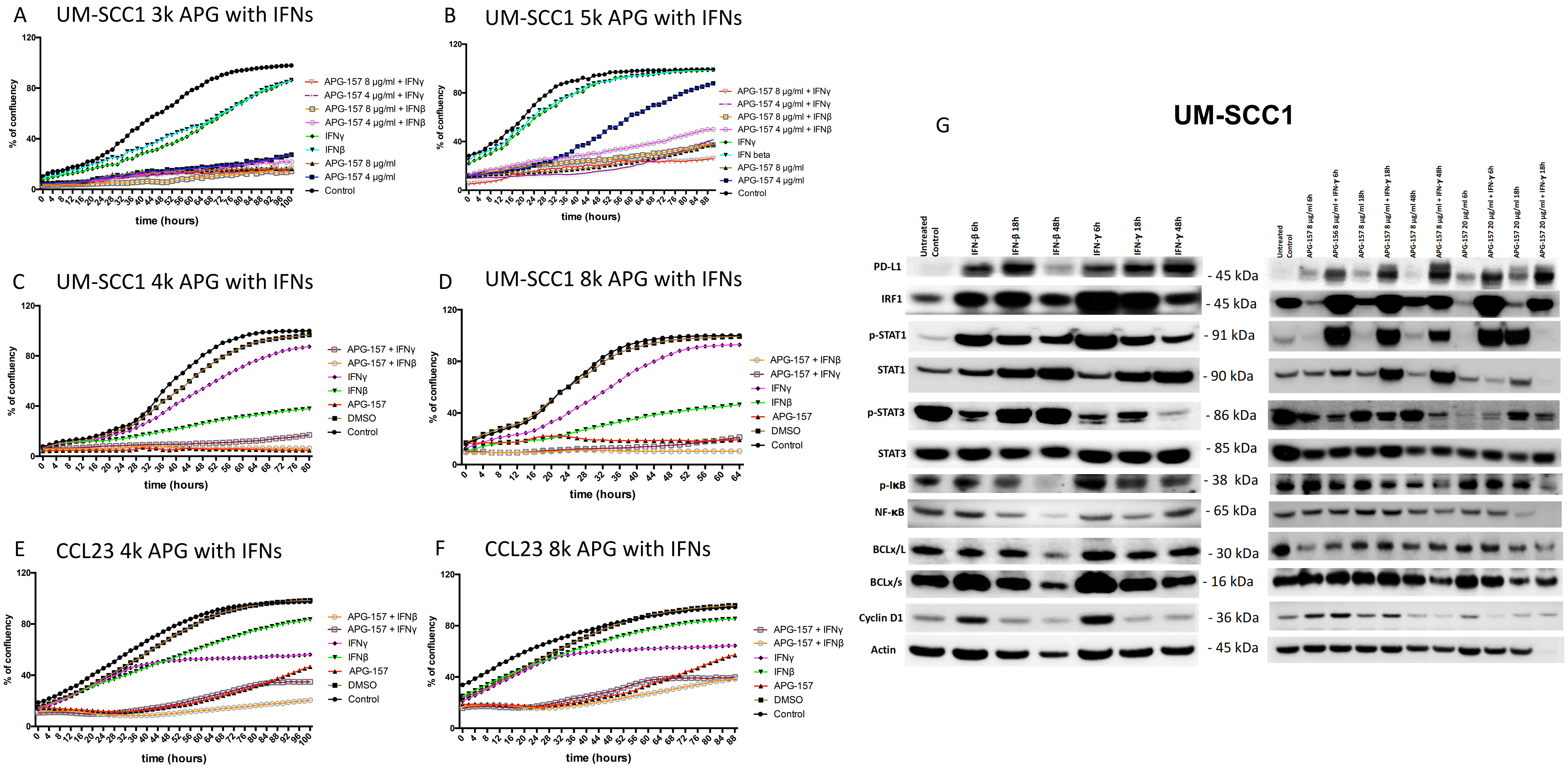

Supplemental Figure S2

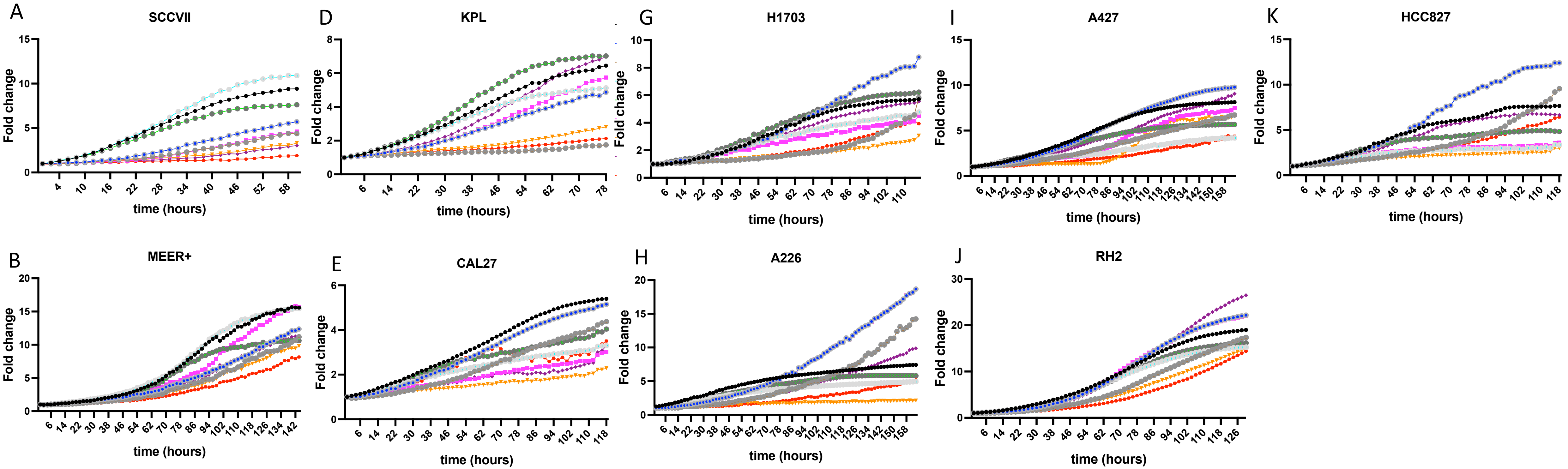

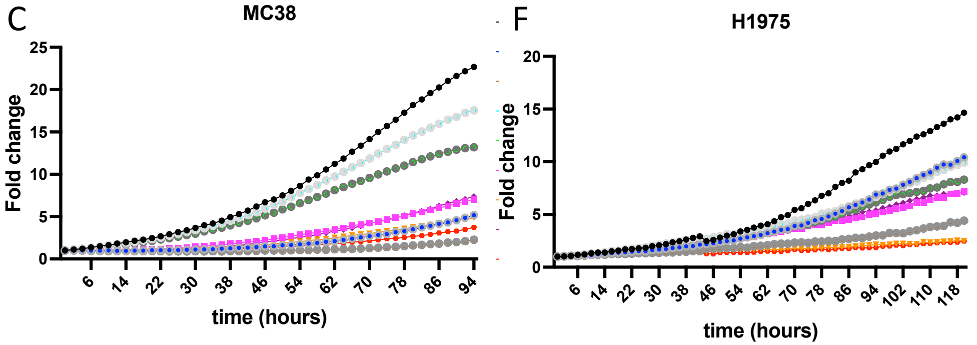

L

	Murine				Human						
	SCCVII	MEER+	MC38	KPL	CAL27	H1975	H1703	A226	A427	RH2	HCC827
Control											
APG-157 4 μg/ml	***	*	***	**							*
APG-157 8 μg/ml	***	**	***	***	***	***	***	*	***	*	**
IFNβ					***		*	***	***		***
IFNγ					*			***	**		***
APG-157 4 μg/ml + IFNβ	***		***	*	***	*	**		***		***
APG-157 8 μg/ml + IFNβ	***	***	***	***	***	***	***	***	***	***	***
APG-157 4 μg/ml + IFNγ	***	*	***		***	*		**			
APG-157 8 μg/ml + IFNγ	***	***	***	***	***	***	***	***	***	***	***

Supplemental Figure S3

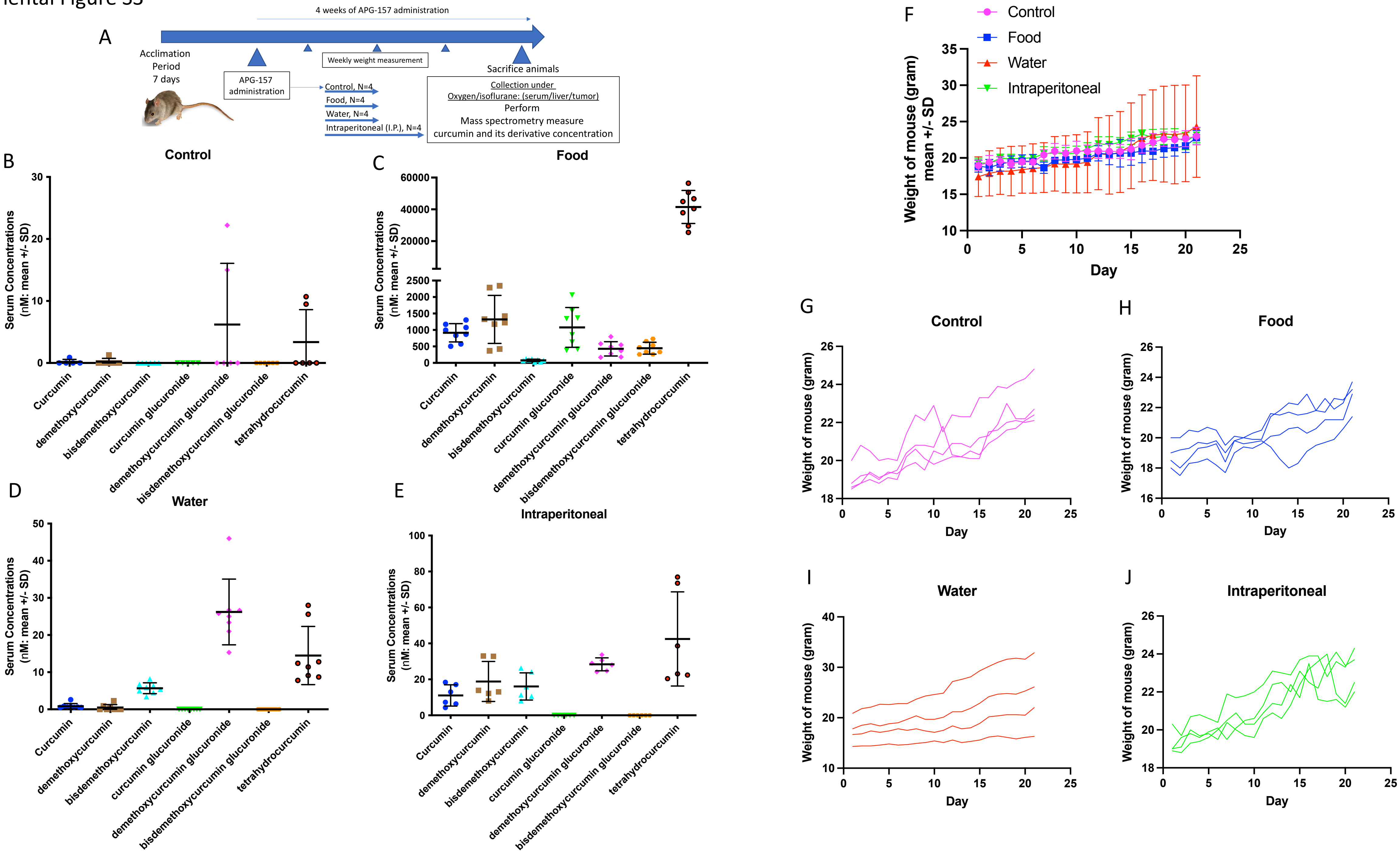

Supplemental Figure S4

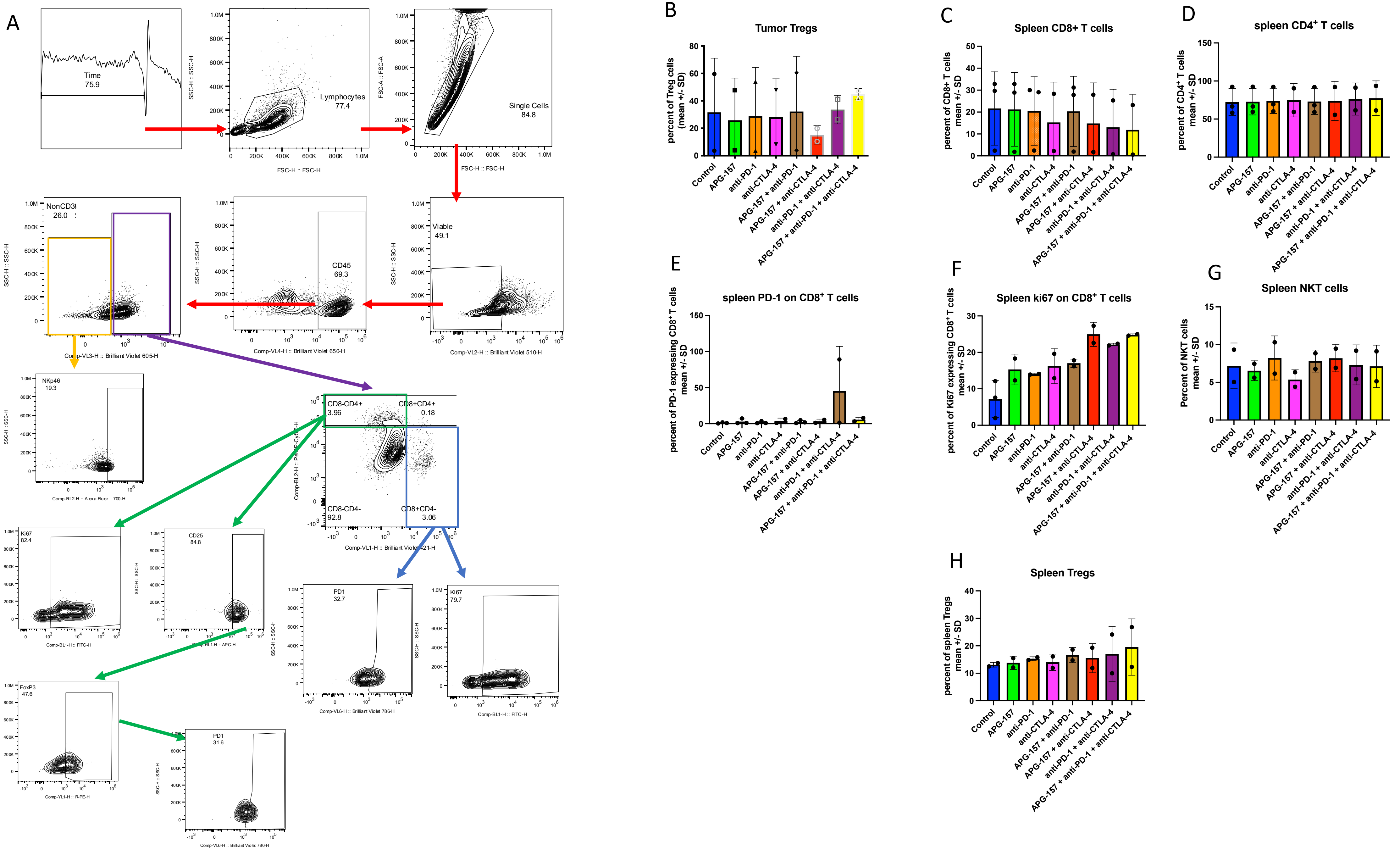

Supplemental Figure S5

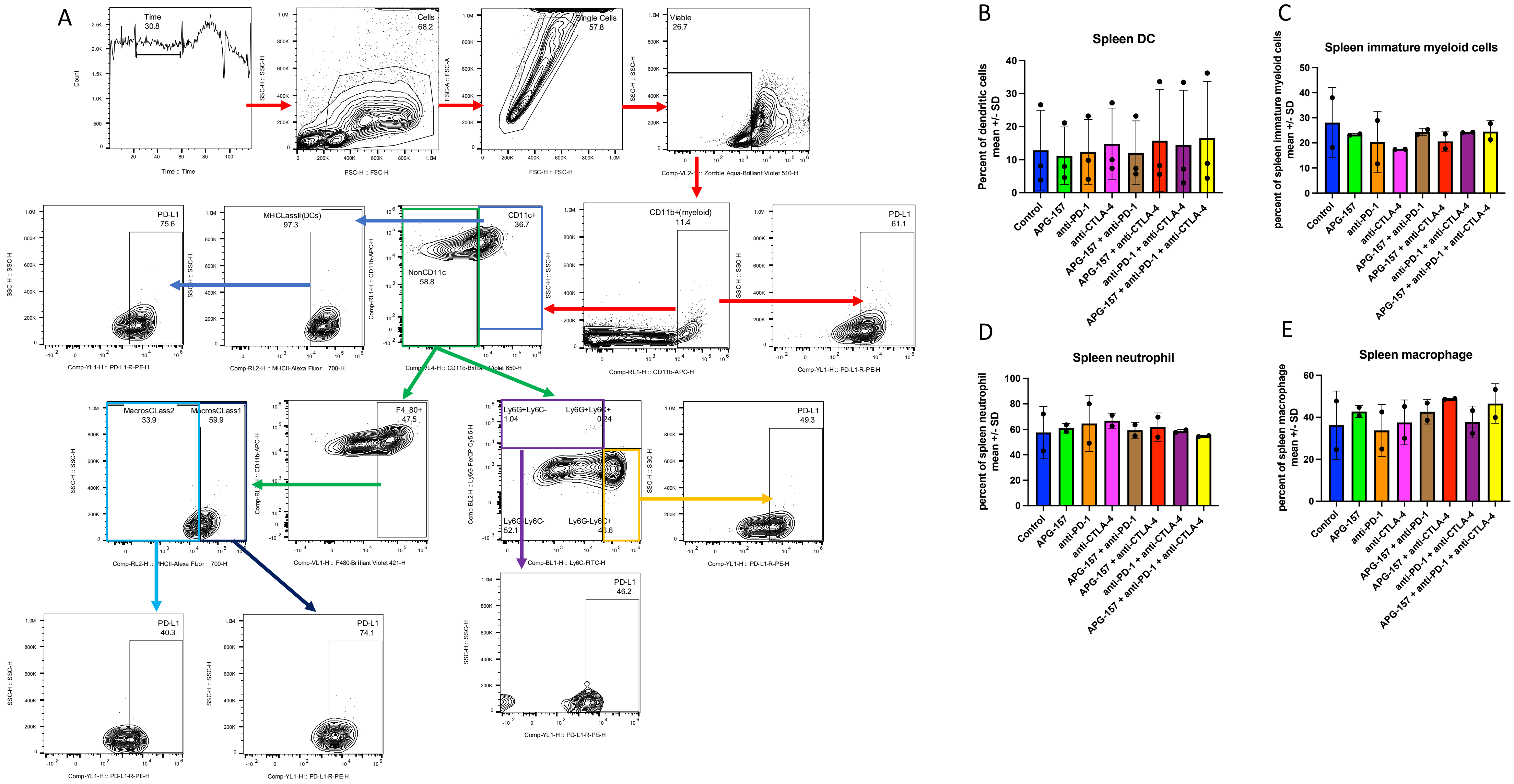

Supplemental Figure S6

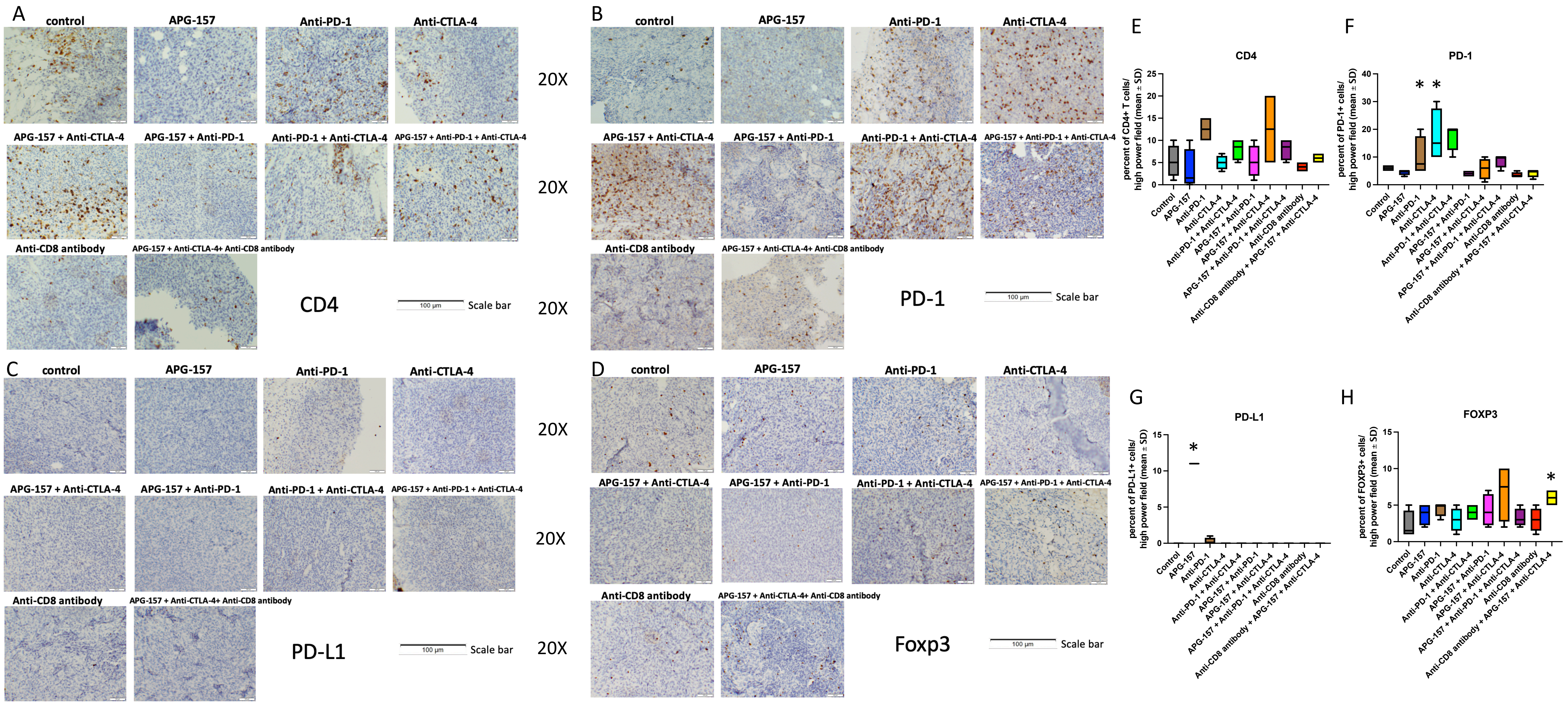

Supplemental Figure S7

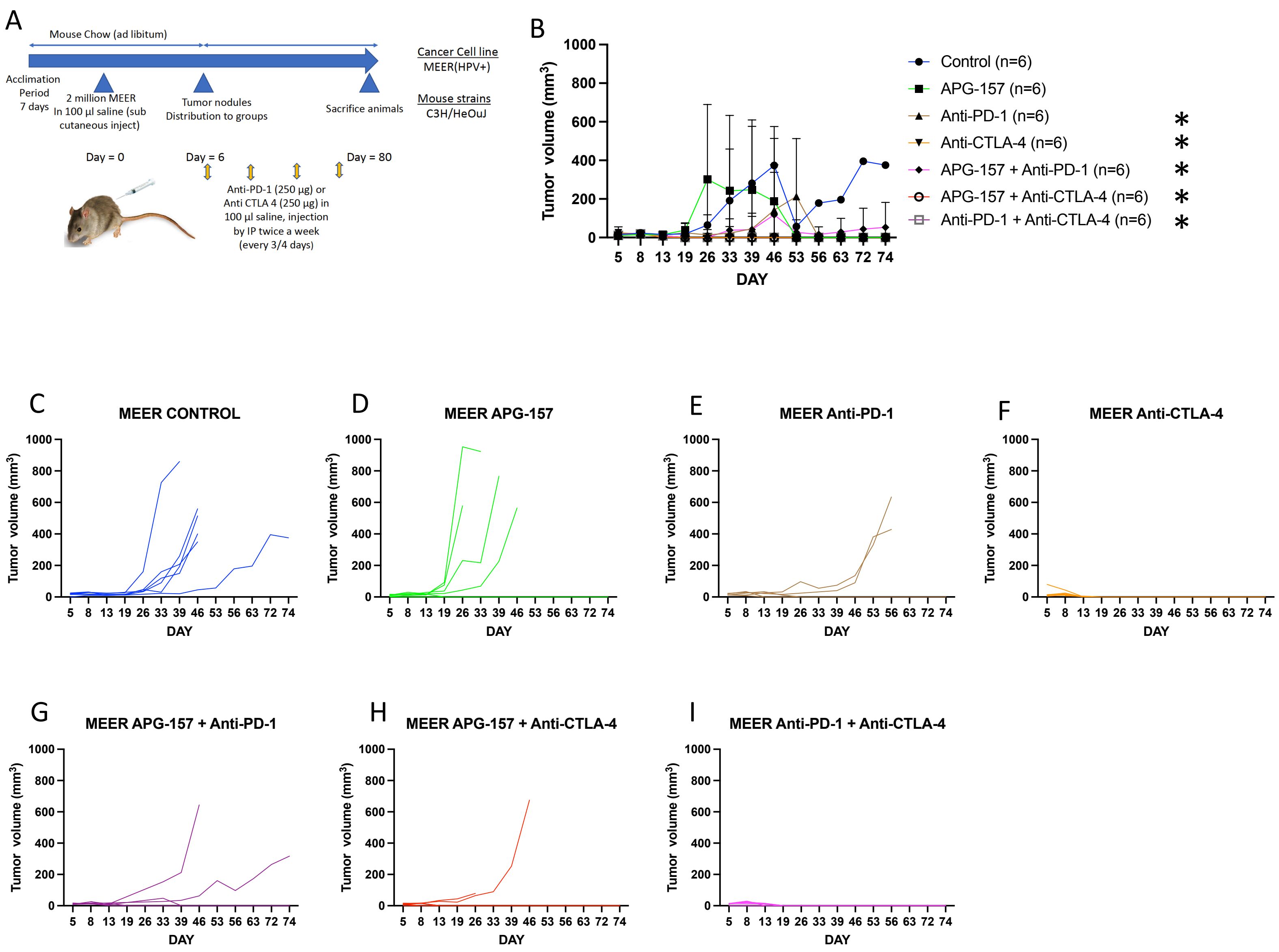

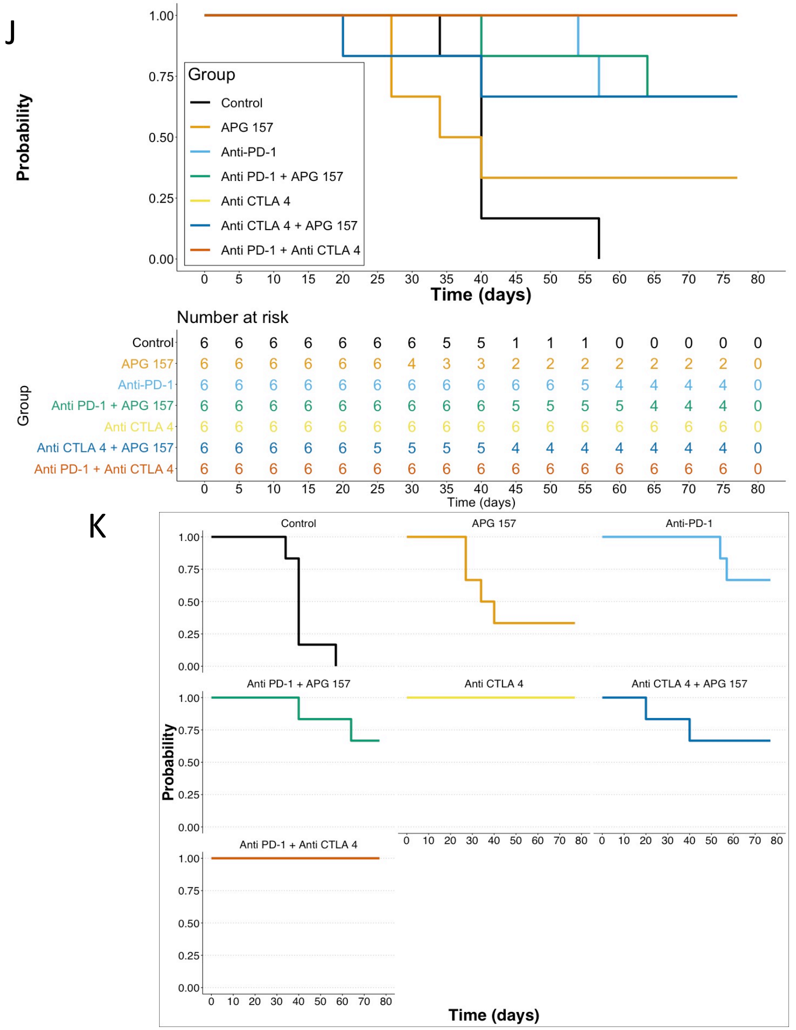

L

Group	HR (95% CI)
Control	-
APG 157	0.62 (0.17-2.29, p=0.476)
Anti-PD-1	0.15 (0.03-0.80, p=0.026)
Anti PD-1 + APG 157	0.16 (0.03-0.82, p=0.028)
Anti CTLA 4	0.00 (0.00-Inf, p=0.998)
Anti CTLA 4 + APG 157	0.19 (0.04-1.00, p=0.049)
Anti PD-1 + Anti CTLA 4	0.00 (0.00-Inf, p=0.998)

Supplemental Figure S8

A

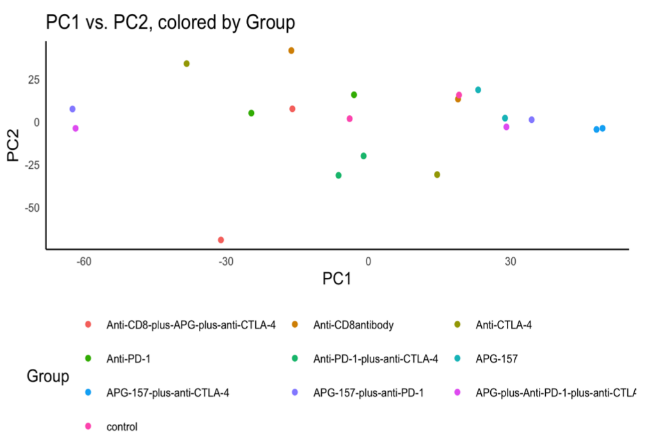

B

Experimental group	Up-regulated	Down-regulated
Anti-CD8	0	4
Anti-CD8 + APG + Anti-CTLA4	116	6
Anti-CTLA4	3	7
Anti-PD1	1	1
Anti-PD1 + Anti-CTLA4	25	2
APG	0	4
APG + Anti-CTLA4	15	46
APG + Anti-PD1	3	3
APG + Anti-PD1 + Anti-CTLA4	2	4

C

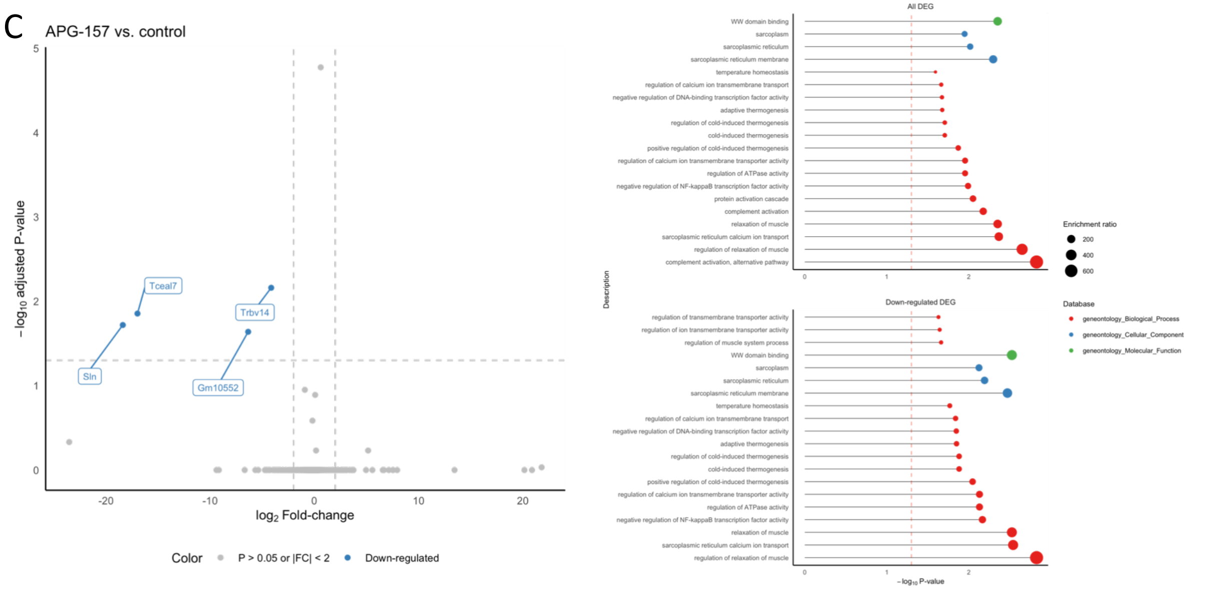

D

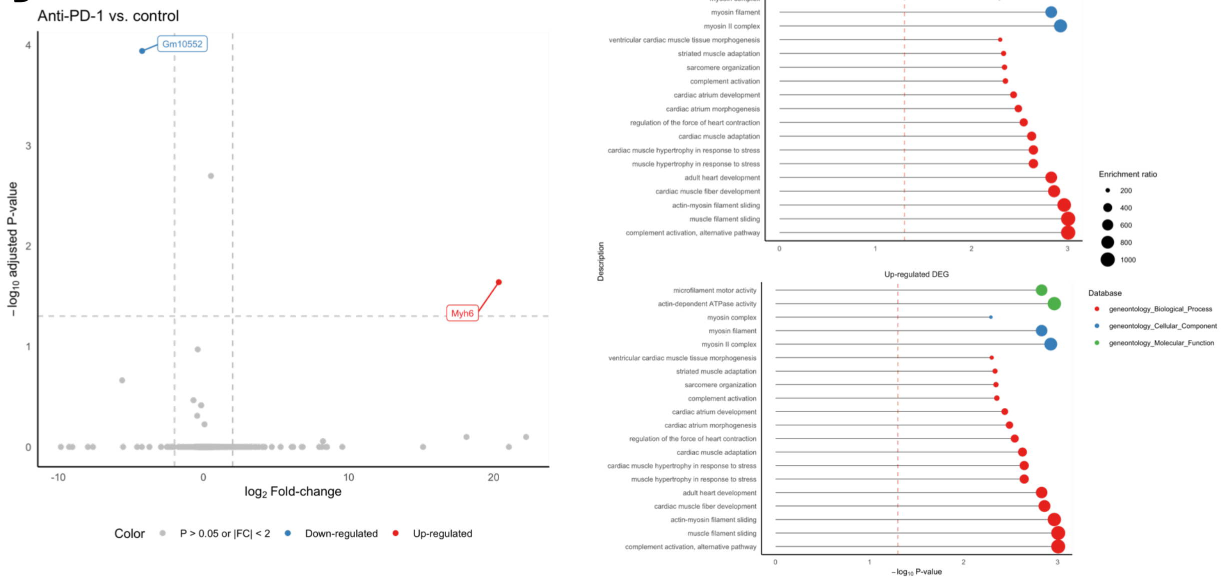

E

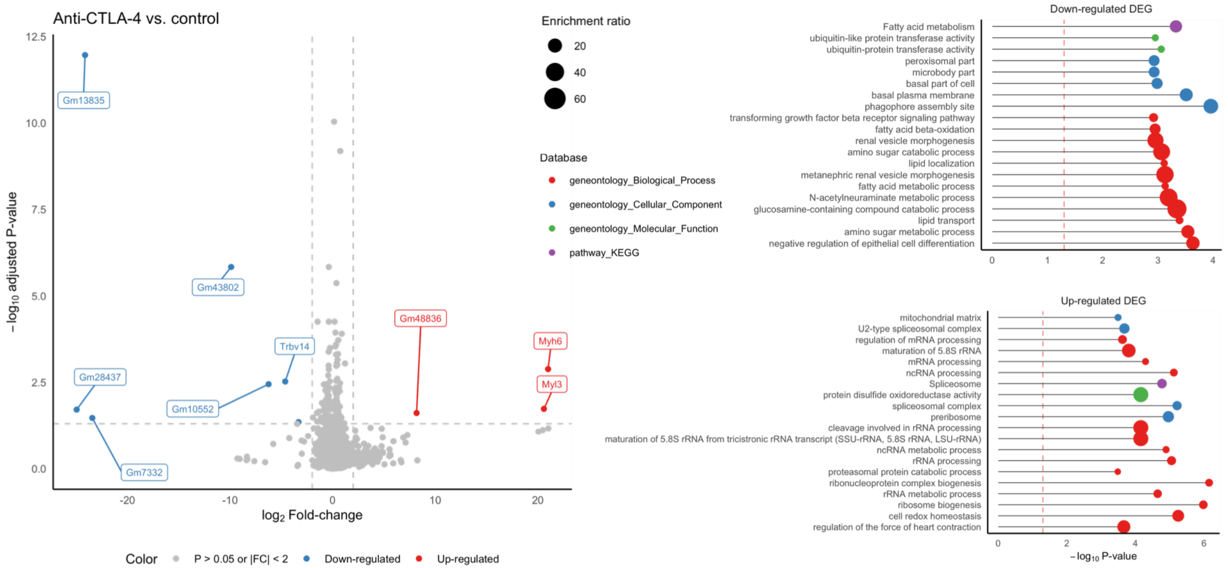

Supplemental Figure S9

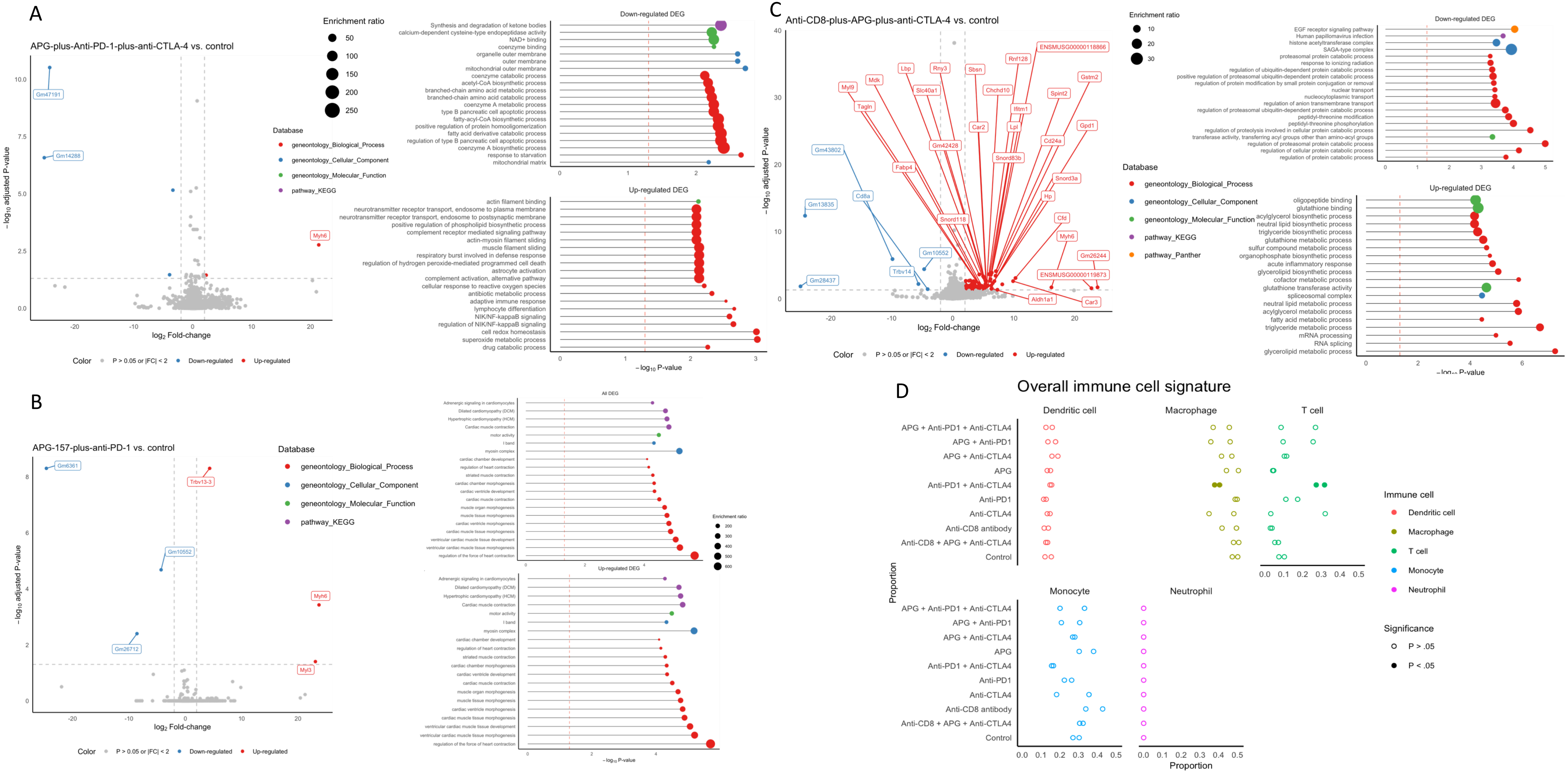

Supplemental Figure S10

A

Ensembl	Gene	Chromosome	Start	End	BaseMean	Log2FC	LFC_SE	Test_Statistic	P	Adjusted_P
ENSMUSG00000061462	Obscn	11	58885082	59029996	351.1243	-21.1383	3.137323	-6.73768	1.61E-11	1.52E-08
ENSMUSG00000072720	Myo18b	5	1.13E+08	1.13E+08	215.5985	-21.036	3.581275	-5.87388	4.26E-09	2.2E-06
ENSMUSG00000029683	Lmod2	6	24597761	24605413	207.0933	-20.8532	3.688002	-5.65434	1.56E-08	5.93E-06
ENSMUSG00000021622	Ckmt2	13	92001506	92025004	231.6912	-19.3792	3.734191	-5.18966	2.11E-07	5.13E-05
ENSMUSG00000025488	Cox8b	7	1.4E+08	1.4E+08	103.6238	-18.8739	3.010043	-6.2703	3.6E-10	2.41E-07
ENSMUSG00000042045	Sln	9	53757448	53761844	50.76722	-18.6295	4.086903	-4.55834	5.16E-06	0.000604
ENSMUSG00000016349	Eef1a2	2	1.81E+08	1.81E+08	471.1963	-17.9375	3.463897	-5.1784	2.24E-07	5.3E-05
ENSMUSG00000079428	Tceal7	X	1.35E+08	1.35E+08	61.79923	-17.2432	3.672605	-4.69509	2.66E-06	0.000356
ENSMUSG00000105373	Gm42429	5	44291735	44292292	3980.306	-13.5196	1.906159	-7.0926	1.32E-12	1.87E-09
ENSMUSG00000106666	Gm43587	3	1.13E+08	1.13E+08	71.34565	-8.83741	2.307864	-3.82926	0.000129	0.00684

B

Obscn in H&N cancer: Not prognostic

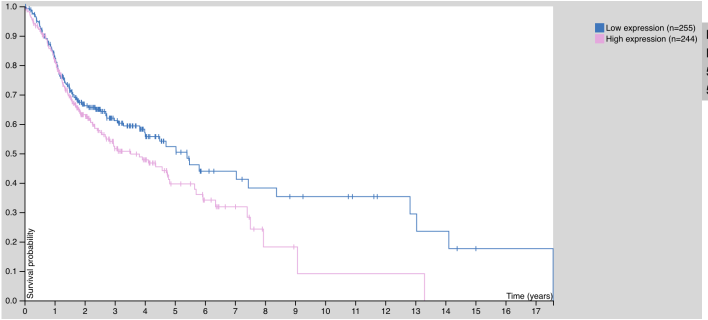

Survival Analysis

Median follow up time :	1.75
P score	0.064
5-year survival high	40%
5-year survival low	52%

C

Myo18b in H&N cancer

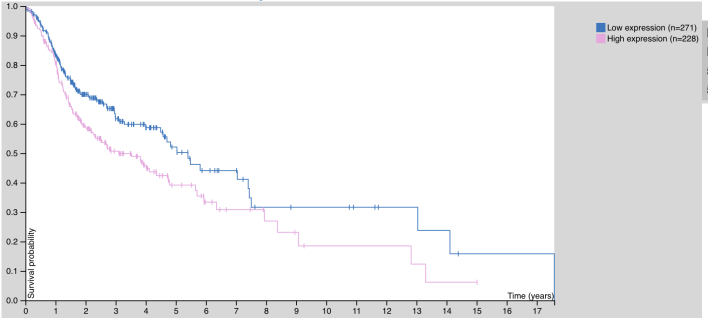

Survival Analysis

Median follow up time :	1.75
P score	0.010
5-year survival high	39%
5-year survival low	52%

D

Ckmt2 in H&N cancer

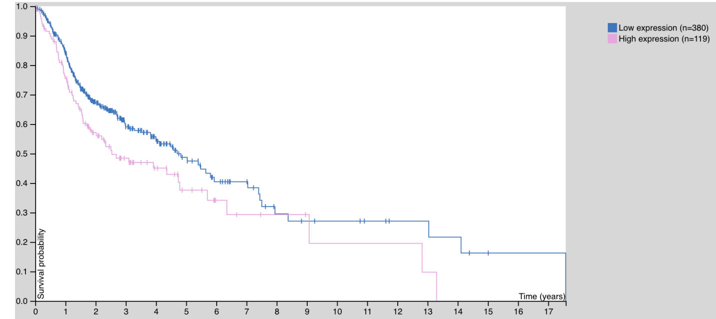

Survival Analysis

Median follow up time :	1.75
P score	0.024
5-year survival high	38%
5-year survival low	49%

E

Sln in renal cancer

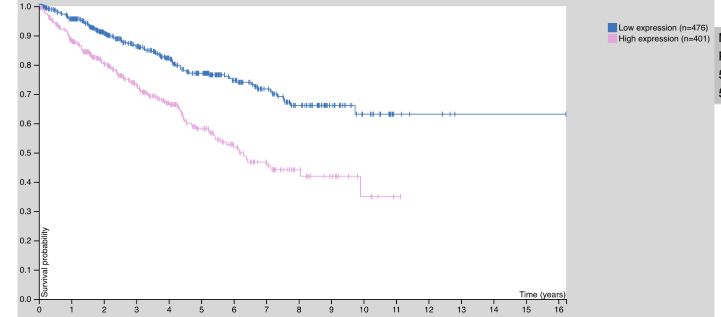

Survival Analysis

Median follow up time :	2.99
P score	3.5e-9
5-year survival high	58%
5-year survival low	77%

D

Eef1a2 in H&N cancer

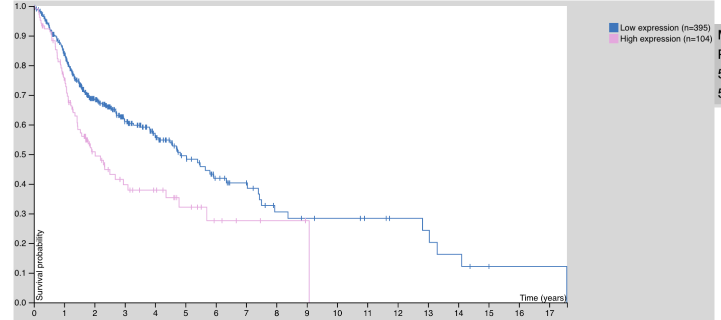

Survival Analysis

Median follow up time :	1.75
P score	0.00091
5-year survival high	32%
5-year survival low	50%

F

Tceal7 in renal cancer

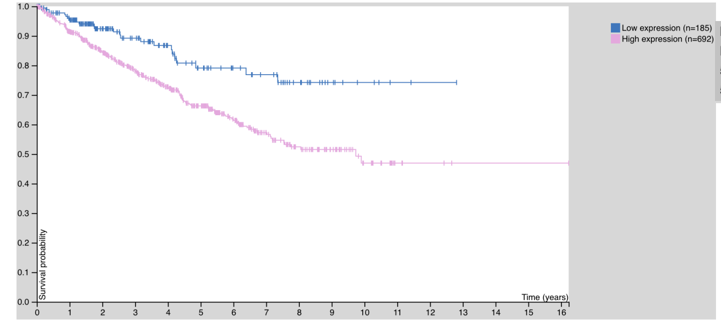

Survival Analysis

Median follow up time :	2.99
P score	0.00043
5-year survival high	66%
5-year survival low	79%

Supplemental Figure S11

A

Ensembl	Gene	Chromosome	Start	End	BaseMean	Log2FC	LFC_SE	Test_Statistic	P	Adjusted_P
ENSMUSG00000111890	Gm48836	10	5041006	5041286	16.77848	7.069151	2.217022	3.18858	0.00143	0.034643
ENSMUSG00000022126	Acod1	14	1.03E+08	1.03E+08	1584.486	3.87535	1.061456	3.650976	0.000261	0.011119
ENSMUSG00000031596	Slc7a2	8	41315433	41375345	492.1371	3.538599	0.940631	3.761943	0.000169	0.008367
ENSMUSG00000076470	Trbv13-3	6	41107081	41107519	19.23158	3.413655	0.612081	5.577129	2.45E-08	8.96E-06
ENSMUSG00000105651	1700017M07Rik	3	35310611	35330280	52.54668	3.411212	0.491658	6.938183	3.97E-12	4.51E-09
ENSMUSG00000049723	Mmp12	9	7344381	7369499	710.6238	3.33364	1.077445	3.094022	0.001975	0.041249
ENSMUSG00000046908	Ltb4r1	14	56003419	56005951	289.6353	3.237709	0.688251	4.704257	2.55E-06	0.000345
ENSMUSG00000023132	Gzma	13	1.13E+08	1.13E+08	385.8003	3.074394	0.717546	4.284593	1.83E-05	0.001576
ENSMUSG00000015437	Gzmb	14	56496295	56499717	1079.507	3.011865	0.872738	3.451052	0.000558	0.019465
ENSMUSG00000037202	Prf1	10	61133612	61140459	1059.255	2.79106	0.871165	3.203823	0.001356	0.033649

B

Mmp12 in H&N cancer: not prognostic

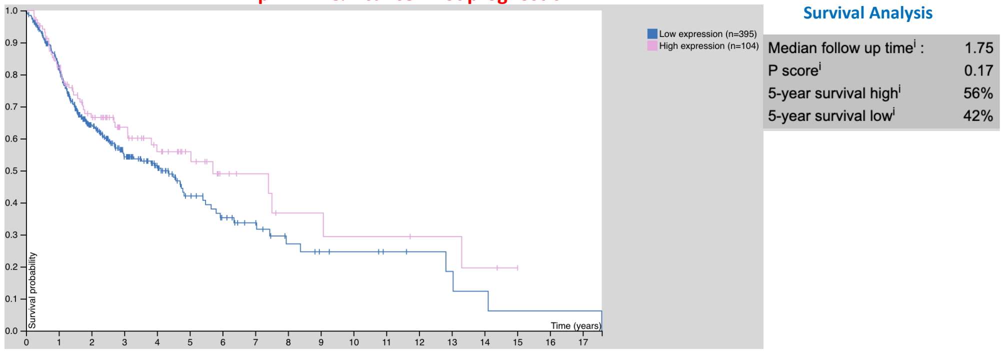

C

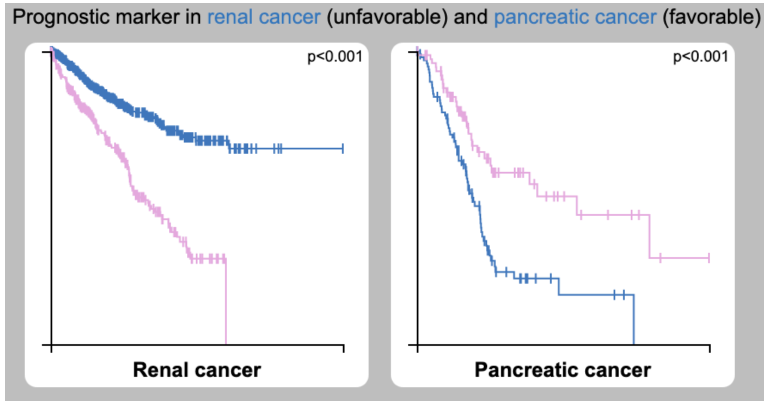

Ltb4r1 in pancreatic cancer

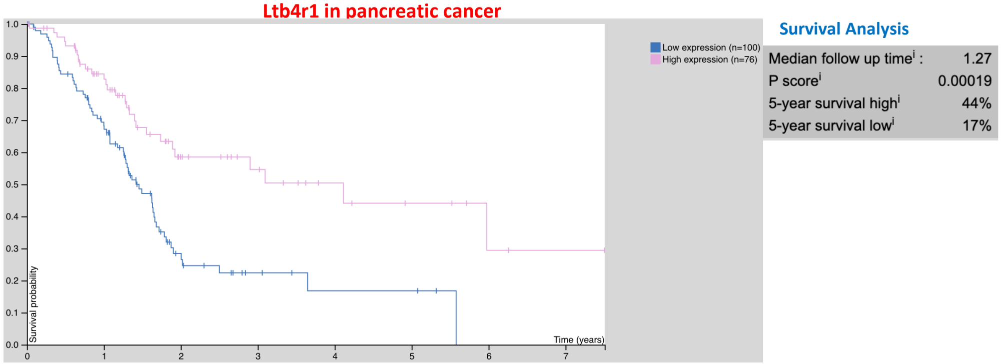

Supplement: Supplementary file 1 — Data S1. [file CAM4-13-e7212-s001.pdf]
